# Deep learning imaging phenotype can classify metabolic syndrome and is predictive of cardiometabolic disorders

**DOI:** 10.1186/s12967-024-05163-1

**Published:** 2024-05-08

**Authors:** Jacob S. Leiby, Matthew E. Lee, Manu Shivakumar, Eun Kyung Choe, Dokyoon Kim

**Affiliations:** 1 Department of Biostatistics, Epidemiology, and Informatics, Perelman School of Medicine, University of Pennsylvania, 19104 Philadelphia, PA USA; 2 Department of Surgery, Seoul National University Hospital Healthcare System Gangnam Center, 06236 Seoul, South Korea; 3 Institute for Biomedical Informatics, Perelman School of Medicine, University of Pennsylvania, 19104 Philadelphia, PA USA

**Keywords:** Metabolic syndrome, Computed tomography, Deep learning, Machine learning

## Abstract

**Background:**

Cardiometabolic disorders pose significant health risks globally. Metabolic syndrome, characterized by a cluster of potentially reversible metabolic abnormalities, is a known risk factor for these disorders. Early detection and intervention for individuals with metabolic abnormalities can help mitigate the risk of developing more serious cardiometabolic conditions. This study aimed to develop an image-derived phenotype (IDP) for metabolic abnormality from unenhanced abdominal computed tomography (CT) scans using deep learning. We used this IDP to classify individuals with metabolic syndrome and predict future occurrence of cardiometabolic disorders.

**Methods:**

A multi-stage deep learning approach was used to extract the IDP from the liver region of unenhanced abdominal CT scans. In a cohort of over 2,000 individuals the IDP was used to classify individuals with metabolic syndrome. In a subset of over 1,300 individuals, the IDP was used to predict future occurrence of hypertension, type II diabetes, and fatty liver disease.

**Results:**

For metabolic syndrome (MetS) classification, we compared the performance of the proposed IDP to liver attenuation and visceral adipose tissue area (VAT). The proposed IDP showed the strongest performance (AUC 0.82) compared to attenuation (AUC 0.70) and VAT (AUC 0.80). For disease prediction, we compared the performance of the IDP to baseline MetS diagnosis. The models including the IDP outperformed MetS for type II diabetes (AUCs 0.91 and 0.90) and fatty liver disease (AUCs 0.67 and 0.62) prediction and performed comparably for hypertension prediction (AUCs of 0.77).

**Conclusions:**

This study demonstrated the superior performance of a deep learning IDP compared to traditional radiomic features to classify individuals with metabolic syndrome. Additionally, the IDP outperformed the clinical definition of metabolic syndrome in predicting future morbidities. Our findings underscore the utility of data-driven imaging phenotypes as valuable tools in the assessment and management of metabolic syndrome and cardiometabolic disorders.

**Supplementary Information:**

The online version contains supplementary material available at 10.1186/s12967-024-05163-1.

## Background

Cardiometabolic disorders, such as type II diabetes and cardiovascular disease, have become a global health concern with significant morbidity and mortality rates [1–4]. Metabolic syndrome, a condition characterized by a clustering of metabolic abnormalities, is a known risk factor for these diseases [5–8]. The overall prevalence of metabolic syndrome in the United States has moderately increased among all adults in the last decade, but has increased significantly among specific groups, including women, young adults, and Asian and Hispanic adults [9]. Clinical definitions of metabolic syndrome vary among organizations, but typically consist of a combination of high blood pressure, dyslipidemia, central obesity, and glucose intolerance [10]. Lifestyle modifications and medications have been shown to effectively mitigate these conditions, reducing the incidence of metabolic syndrome [11–13]. Therefore, early detection and intervention for individuals with metabolic abnormalities may help reduce the risk of developing more serious metabolic-related diseases.

Image-derived phenotypes (IDPs) are quantitative measurements extracted from medical imaging that are used as potential biomarkers for disease. Traditionally, hand-crafted features based on prior expert knowledge, such as volume, activation, or attenuation of regions of interest are extracted to overcome the high dimensionality of imaging data. IDPs from brain imaging modalities have shown strong associations with morbidity and genetic risk for Alzheimer’s disease [14–16]. Studies have also explored IDPs from computed tomography (CT) scans and magnetic resonance imaging and their associations with metabolic-related diseases [17–19]. In particular, hand-crafted features including abdominal fat and muscular measurements, aortic calcium quantification, and volumetric liver attenuation acquired from abdominal CT scans were found to be strongly associated with metabolic syndrome and subsequent cardiovascular events [18, 20]. Unenhanced abdominal CT scans are a particularly favorable modality for extracting IDPs as they are performed for many indications and allow clinicians to extract additional biologically relevant information in a non-invasive manner.

Hand-crafted radiomic features, such a volumetric measurements, are efficient at converting high-dimensional data into simple representations that can be used for disease association or classification. However, imaging data consists of rich information, and these hand-crafted features rely on previous knowledge and may miss biologically relevant signal. Recent advances in machine learning have enabled data-driven approaches to derive IDPs using deep learning-based models to that are able to condense high-dimensional data into compact representations of unobservable information. Data-driven IDPs have been proposed in a variety of settings for both population-based and disease-specific phenotypes [21–25]. 

In this study, we aimed to develop a deep learning-derived IDP of metabolic abnormality and use it to predict future occurrence of cardiometabolic disorders. To do this, we treated the clinical definition of metabolic syndrome as a proxy of metabolic abnormality and trained a deep learning model to classify it from unenhanced abdominal CT scans. We focused on three-dimensional (3D) segmented liver regions to directly compare our results to the performance of liver attenuation (expressed as Hounsfield units), a hand-crafted feature that is linearly correlated with hepatic fat and is highly associated with metabolic syndrome [18, 26–28]. Additionally, we included comparisons to visceral adipose tissue area (VAT), another traditional IDP strongly associated with metabolic syndrome [18]. Our IDP demonstrated significant superiority over attenuation and VAT in classifying metabolic syndrome. Furthermore, we performed an association analysis of the IDP with 115 clinical phenotypes and observed significant associations with anthropometric measurements and endocrine, metabolism, and digestive phenotypes. Lastly, we used the IDP to predict the future occurrence of metabolic-related morbidities. Our findings revealed that the IDP significantly enhanced the prediction of fatty liver disease and type II diabetes compared to relevant baseline covariates and the clinical definition of metabolic syndrome and performed comparably for prediction of hypertension.

## Methods

### Data and clinical definitions

#### Study population

For the model development, we used data from a comprehensive health check-up cohort of a Korean population consisting of 2,268 individuals (Table 1). The details of the dataset are described elsewhere [29]. Briefly, at the Seoul National University Hospital Gangnam Center, individuals participate in comprehensive health screening for anthropometric, cardiovascular, digestive, endocrinological, metabolic, hematologic, lung, and renal conditions with various clinical tests including abdominal computed tomography (CT) scans. For 1,397 individuals, we collected the 5-year follow up data (Supplementary Table 1). For external replication of the developed model, we used data and CT scans of preoperative evaluation tests from stage I colorectal cancer patients, who underwent curative colectomy in Seoul National University hospital [30]. We used the non-contrast (unenhanced) abdominal CT scan stored in Digital Imaging and Communications in Medicine (DICOM) file format. Abdominal CT scans were performed using a 64-slice multi-detector CT scanner (Brilliance 64 scanners; Philips Healthcare, Amsterdam, Netherlands) with 3 mm thickness and increment.

**Table 1:** Characteristics of the baseline development cohort [29]

	Metabolic syndrome (-)	Metabolic syndrome (+)	P value	N
Baseline Age	53.4 ± 8.4	55.0 ± 8.0	<0.001	2268
Year of baseline enrollment			0.203	2268
2014	1607 (90.1%)	430 (88.8%)		
2015	145 (8.1%)	49 (10.1%)		
2016	32 (1.8%)	5 (1.0%)		
Gender			<0.001	2268
Female	636 (35.7%)	74 (15.3%)		
Male	1148 (64.3%)	410 (84.7%)		
Total fat amount (mm2)	25185.2 ± 8438.1	34310.8 ± 8739.1	<0.001	2268
Visceral fat amount (mm2)	10577.3 ± 4873.6	16508.3 ± 4947.0	<0.001	2268
Hypertension diagnosis			<0.001	2268
No	1527 (85.6%)	245 (50.6%)		
Yes	257 (14.4%)	239 (49.4%)		
Hypertension medication			<0.001	2268
No	1576 (88.3%)	273 (56.4%)		
Yes	208 (11.7%)	211 (43.6%)		
Diabetes diagnosis			<0.001	2268
No	1715 (96.1%)	407 (84.1%)		
Yes	69 (3.9%)	77 (15.9%)		
Diabetes medication			<0.001	2268
No	1738 (97.4%)	431 (89.0%)		
Yes	46 (2.6%)	53 (11.0%)		
Dyslipidemia diagnosis			<0.001	2268
No	1500 (84.1%)	353 (72.9%)		
Yes	284 (15.9%)	131 (27.1%)		
Dyslipidemia medication			<0.001	2268
No	1609 (90.2%)	397 (82.0%)		
Yes	175 (9.8%)	87 (18.0%)		
Systolic blood pressure	114.7 ± 12.5	123.4 ± 12.8	<0.001	2263
Diastolic blood pressure	75.7 ± 9.6	82.5 ± 9.2	<0.001	2263
Height	166.7 ± 7.7	169.6 ± 6.9	<0.001	2247
Weight	64.0 ± 10.2	75.4 ± 10.3	<0.001	2246
Body mass index	22.9 ± 2.6	26.1 ± 2.7	<0.001	2260
In body skeletal muscle mass	26.6 ± 5.5	30.4 ± 5.0	<0.001	2244
In body fat mass	16.0 ± 4.5	21.1 ± 5.2	<0.001	2244
In body Fat percent	25.1 ± 6.2	28.0 ± 5.4	<0.001	2244
Waist circumference	82.3 ± 7.3	92.1 ± 6.8	<0.001	2245
Glucose	95.9 ± 13.6	112.1 ± 19.7	<0.001	2246
Triglycerides	95.0 ± 53.4	176.8 ± 96.1	<0.001	2242
HDL cholesterol	54.8 ± 12.0	45.9 ± 9.6	<0.001	2242
HBA1C	5.6 ± 0.5	6.0 ± 0.8	<0.001	2251
Metabolic risk: waist circumference			<0.001	2245
No	1478 (83.9%)	122 (25.3%)		
Yes	284 (16.1%)	361 (74.7%)		
Metabolic risk: Triglycerides			<0.001	2242
No	1584 (90.1%)	185 (38.3%)		
Yes	175 (9.9%)	298 (61.7%)		
Metabolic risk: HDL cholesterol			<0.001	2242
No	1569 (89.2%)	289 (59.8%)		
Yes	190 (10.8%)	194 (40.2%)		
Metabolic risk: glucose			<0.001	2249
No	1273 (72.1%)	81 (16.7%)		
Yes	492 (27.9%)	403 (83.3%)		
Metabolic risk: hypertension			<0.001	2268
No	1285 (72.0%)	102 (21.1%)		
Yes	499 (28.0%)	382 (78.9%)		

Extended information available in Supplementary Table 2. P-values are from t-test for continuous variables and the Fisher exact test for categorical variables

#### Clinical assessment and definitions

All participants entered the test after at least ten hours of fasting. For the definition of metabolic syndrome, we used the criteria suggested by the American Heart Association [31]. In the population for model development, metabolic syndrome was defined when at least three of the following metabolic risk factors were positive: increased waist circumference (male > 102 cm, female > 88 cm); triglycerides > = 150 mg/dL; decreased high-density lipoprotein (HDL) cholesterol (male < 40 mg/dL, female < 50 mg/dL); fasting glucose > = 100 mg/dL or on medication for hyperglycemia; and blood pressure > = 130/85 mmHg or on medication for hypertension, resulting in sixteen possible combinations. Among individuals with metabolic syndrome, the most common contributing factors were increased blood glucose, blood pressure, and waist circumference; elevated blood glucose was the most common component overall (Supplementary Fig. 1). In the validation population from colorectal cancer patients, since the waist circumference, HDL cholesterol, triglyceride data were not available, we used a modified criteria for metabolic syndrome, indirectly; at least three of the following metabolic risk factors were positive: increased body mass index > = 25 kg/m2; on medication of dyslipidemia; fasting glucose > = 100 mg/dL or on medication for hyperglycemia; and blood pressure > = 130/85 mmHg or on medication for hypertension. The visceral adipose tissue area was measured at the level of the umbilicus of the abdominal CT scan, as previously described [32]. 

For all follow up analyses, we defined hypertension and type II diabetes as “on medication for the disease”, and abdominal ultrasound was used to diagnose fatty liver. The abdominal ultrasound procedure was performed by an experienced radiologist. The definition of fatty liver was based on the vascular blurring, attenuation, hepatorenal echo contrast and liver brightness in ultrasonography image [33]. We classified as “normal liver” versus “at least mild degree of fatty liver”, annotated by radiologists.

### Liver segmentation in abdominal CT scans

#### Abdominal CT preprocessing

All DICOM series were converted to 3D Neuroimaging Informatics Technology Initiative (NIfTI) file format using the *dicom2nifti* python package. 3D volumes were preprocessed using the nnU-Net software [34]. Briefly, the intensity values are clipped at the 0.5 and 99.5 percentiles, and each volume is resampled to the median values of the training data spacing in each axis.

#### Segmentation model architecture and optimization

The segmentation model followed the U-Net architecture (Supplementary Fig. 2a) [34]. This model, popular in medical imaging segmentation, adopts an encoder-decoder structure aiming to learn a dense embedding of the input data (encoder) and localize the relevant information back into the input dimensions through an expanding path (decoder). We again used the nnU-Net software to optimize the network topology and the input patch size [34]. The final patch size was set to 28 × 256 × 256.

#### Training and validation

The segmentation model was trained and validated on 358 abdominal CT scans with the liver regions annotated. The annotations were generated by a technician using a commercially available segmentation software program (AVIEW Modeler, version 1.1.42, Corelinesoft Co. Ltd., Seoul, South Korea). We performed 5-fold cross validation and used the Dice coefficient as the performance metric.

The top-performing segmentation model was used to create liver segmentation masks for all remaining samples.

### Image-derived phenotypes

#### Liver attenuation

Liver attenuation values were extracted by applying the mask to the original CT volume and calculating the median attenuation of the voxels predicted as liver.

#### Visceral adipose tissue area

Visceral adipose tissue area was measured at the level of the umbilicus of the abdominal CT scan, as previously described [32]. We defined the visceral adipose tissue are as the adipose tissue area, in parameter cm2, located intra-abdominally, confined by the parietal peritoneum [35]. 

#### Deep learning image-derived phenotype

To derive the imaging phenotype, we initially masked the original volumes, selecting only the predicted liver region. Next, we employed a multi-stage deep learning pipeline [36]. First, we used the pre-trained encoder from the segmentation model to extract a latent representation of the data. Specifically, we performed global average pooling over each encoder block output to aggregate the spatial information and concatenated the pooled embeddings (Supplementary Fig. 2b). Second, we input these embeddings into a neural network classifier with a single node output to classify metabolic syndrome using the binary cross-entropy loss function. (Supplementary Fig. 2c). More details on the model architecture are described in Supplementary Fig. 2. As the segmentation model was optimized for patch-based input, each input volume was divided into $$ X$$ three-dimensional patches, and a latent representation was created for each patch. The classifier incorporated a learned gated attention-pooling function to aggregate the patch information into a volume-level embedding [37]. The classifier was trained using the AdamW optimizer in PyTorch version 1.12.1 with default parameters. To validate the deep learning IDP, we used stratified five-fold cross validation repeated ten times for a total of fifty models. For each split we reserved 20% of the total dataset for testing and evaluation and with the remaining data, trained on 80% and used 20% as a validation set to tune the hyperparameters.

For the metabolic syndrome classification, the final hidden layer of the classifier, containing 256 nodes, was extracted as the IDP. For the phenotypic association and follow-up analyses, the output of the classifier was used as a condensed version of the IDP.

#### Baseline metabolic syndrome classification

We employed elastic-net logistic regression to incorporate the IDP with clinical covariates to predict metabolic syndrome using the R package glmnet version 4.1-1. Specifically, we used the default parameters of the *cv.glmnet* function to perform ten-fold cross-validation in training datasets only and used the optimal hyper parameters to evaluate the performance in the hold-out testing datasets (the validation sets used in hyperparameter tuning for IDP development are not used in these analyses). All numerical features were centered and scaled based on the training dataset means and standard deviations.

### Phenome-wide association analysis

We conducted linear regression for each phenotype of interest, correcting for age and sex in R version 4.1.0. All reported p-values were corrected for multiple-testing using the Bonferroni correction.

### Follow-up disease analyses

We conducted predictive analyses for three metabolic-related morbidities—hypertension, fatty liver disease, and type II diabetes. For each disease, we removed all individuals at baseline who already had the respective condition, extracted baseline condensed IDPs for the remaining individuals, and collected baseline metabolic syndrome status and covariate measurements. We compared models that included relevant baseline covariates, clinically defined metabolic syndrome, and the IDP.

We used the same training splits as in the previous analyses to fit a logistic regression, and then used that model to predict disease status in the hold-out datasets (the validation sets used in hyperparameter tuning for IDP development are not used in these analyses). All numerical features were centered and scaled based on the training dataset means and standard deviations.

To classify individuals as metabolically abnormal at baseline, the optimal cutoff value of the IDP was determined as the threshold value that maximized the sensitivity and specificity [38]. 

### Performance evaluation and statistical tests

We employed the area under the receiver operating characteristic curve (AUC) and area under the precision-recall curve (AURPC) as measures of model performance. A paired *t*-test was used to compare the AUCs between different models.

For statistical tests comparing demographic and clinical information between metabolic syndrome positive and negative cases, we used the *t*-test for continuous variables and the Fisher exact test for categorical variables in R version 4.1.0.

## Results

### IDP accurately classifies baseline metabolic syndrome

**Fig. 1:**
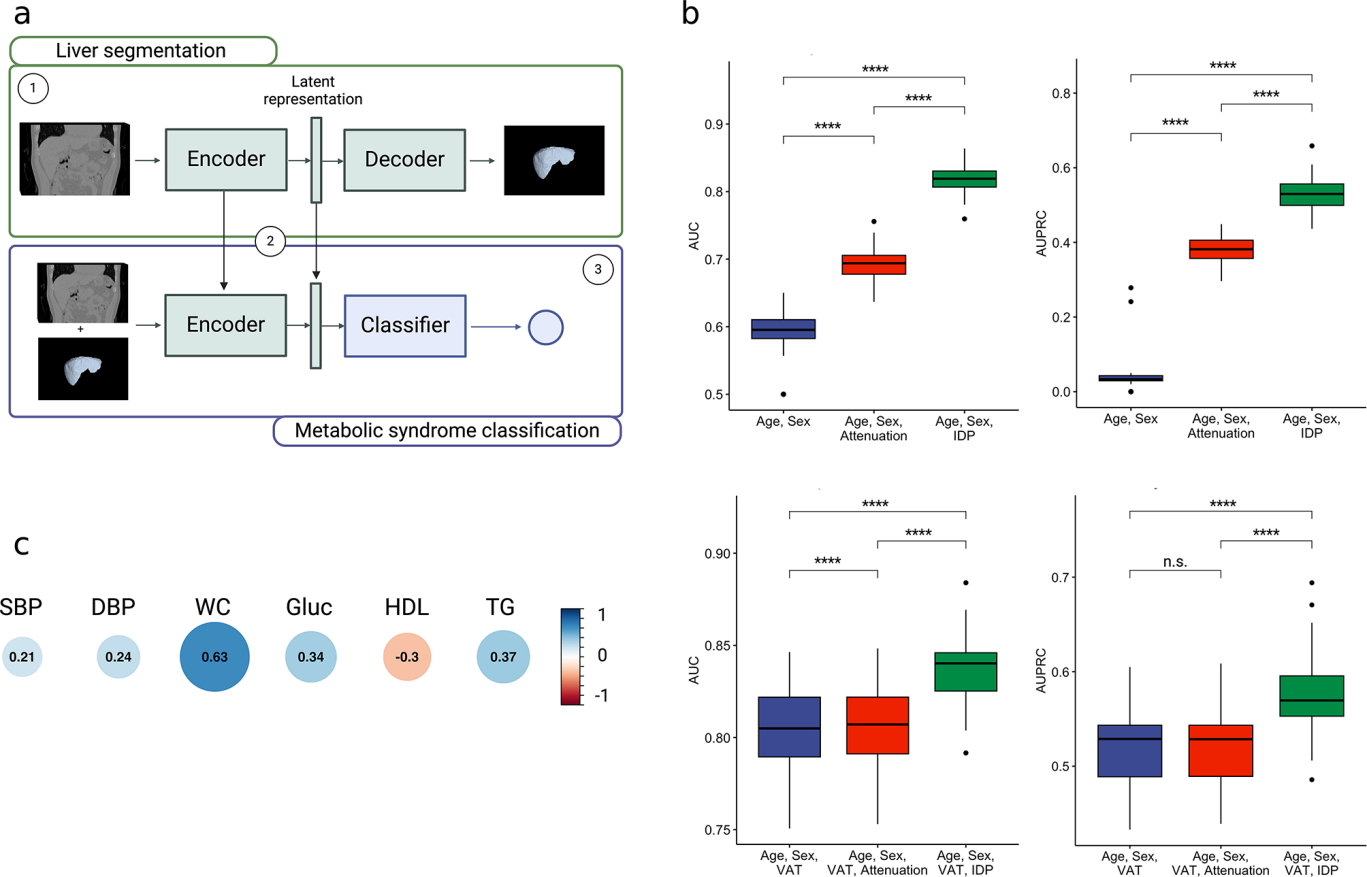
**a**, Overview of three-stage IDP development model: Stage 1 - Training the 3D liver segmentation model; Stage 2 - Using the pretrained encoder to extract latent representations from input CT scans; Stage 3 - Training the metabolic system classifier. **b**, Predictive performance comparing covariates and attenuation to the IDP. **c**, Correlation between the IDP and metabolic syndrome risk factors. IDP, image-derived phenotype; AUC, area under receiver-operating curve; Attenuation, median volumetric liver attenuation (expressed as Hounsfield units); VAT, visceral adipose tissue; SBP, systolic blood pressure; DBP, diastolic blood pressure; WC, waist circumference; Gluc, glucose; HDL, high-density lipoprotein; TG, triglycerides; **** *P* < 0.0001, n.s. not significant, paired t-test

To develop the image-derived phenotype (IDP) of metabolic abnormality, we constructed a multi-stage deep learning pipeline that involved segmentation of the liver region from 3D unenhanced CT scans followed by training a metabolic syndrome classifier on the segmentation model embeddings (Fig. 1a, Methods). The liver segmentation model was trained on 358 manually annotated CT scans and achieved a Dice score of 0.98 through five-fold cross-validation. The final hidden layer of the trained metabolic syndrome classifier is extracted as the IDP. (The classification network achieved a mean AUC of 0.81 (0.02) and AUPRC of 0.52 (0.05)). Using regularized logistic regression to classify metabolic syndrome, the IDP achieved an AUC of 0.82 through ten times repeated five-fold cross-validation compared to an AUC of 0.69 using extracted volumetric liver attenuation, an AUC of 0.80 using visceral adipose tissue area (VAT), and an AUC of 0.59 using age and sex (Fig. 1b, top, Table 2, and Methods). Additionally, we assessed the utility of our model in combination with VAT. When including VAT as an additional covariate the model achieved an AUC of 0.84, suggesting complementary information between the two IDPs (Fig. 1b, bottom, and Table 2).

The output of the deep learning model exhibited significant correlations with each of the individual metabolic syndrome components (Fig. 1c). The direction of these correlations aligns with the definition of metabolic syndrome, with the strongest correlations observed for waist circumference and lipid levels. Many studies have previously shown the associations between waist circumference and lipid levels with liver diseases [39–41]. 

**Table 2:** Performance metrics for metabolic syndrome classification, mean and standard deviation

Cohort	Features	AUC	AUPRC
Development	Age, Sex	0.589 (0.038)	0.041 (0.044)
	Age, Sex, Attenuation	0.693 (0.025)	0.381 (0.034)
	Age, Sex, IDP	0.817 (0.020)	0.531 (0.046)
	Age, Sex, VAT	0.804 (0.023)	0.518 (0.043)
	Age, Sex, VAT, Attenuation	0.805 (0.023)	0.520 (0.043)
	Age, Sex, VAT, IDP	0.837 (0.019)	0.574 (0.042)
External	Age, Sex	0.542 (0.017)	0.067 (0.039)
	Age, Sex, Attenuation	0.618 (0.005)	0.267 (0.002)
	Age, Sex, IDP	0.664 (0.007)	0.321 (0.010)

*IDP* image-derived phenotype; *AUC* area under receiver-operating curve; *AUPRC* area under precision-recall curve; Attenuation, median volumetric liver attenuation (expressed as Hounsfield units); *VAT* visceral adipose tissue

To validate the IDP, we predicted metabolic syndrome in 521 individuals from an external cohort (Supplementary Table 3). For this analysis, we used a modified definition of metabolic syndrome due to data availability (Methods). The IDP significantly outperformed covariate models, achieving an AUC of 0.66 compared to AUC of 0.62 and 0.54 for liver attenuation and age and sex, respectively (Supplementary Fig. 3 and Table 2). This decrease in performance compared to the development dataset may be due to the underlying disease profiles and imaging characteristics of the external dataset.

These results suggest that our framework learns a strong IDP for metabolic abnormality. Through cross-validation, it can better classify metabolic syndrome versus existing biomarkers and covariates and this trend holds when applied to an external validation cohort.

### IDP shows sex-specific differences

**Fig. 2:**
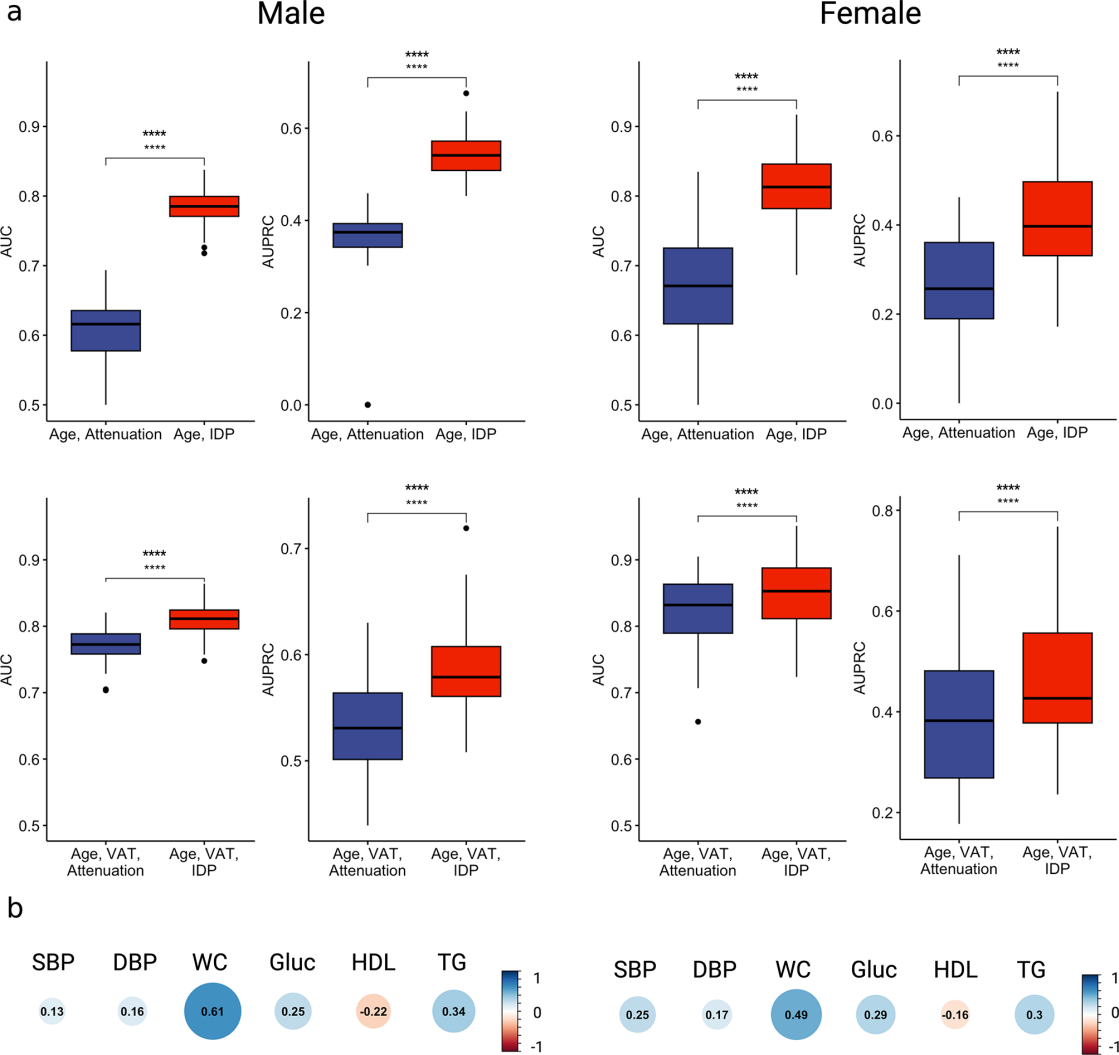
**a**, Predictive performance comparing covariate models to the IDP, stratified by sex. (Male, left column. Female, right column). **b**, Correlation between the IDP and metabolic syndrome risk factors. IDP, image-derived phenotype; AUC, area under receiver-operating curve; Attenuation, median volumetric liver attenuation (expressed as Hounsfield units); VAT, visceral adipose tissue; SBP, systolic blood pressure; DBP, diastolic blood pressure; WC, waist circumference; HDL, high-density lipoprotein; TG, triglycerides; **** *P* < 0.0001, paired t-test

To investigate potential variations in the IDP between sex, we performed stratified analyses. Males have a metabolic syndrome prevalence rate of 26% (410/1558) and females have a prevalence of 10% (74/710). We found that the IDP significantly improves prediction of metabolic syndrome in both males and females (Fig. 2; Table 3). Additionally, incorporating VAT with our IDP yielded improved performance in both sexes compared to a model utilizing VAT alone. Interestingly, for males the IDP alone achieved a higher AUC than VAT and for females VAT outperformed the IDP (Table 3).

**Table 3:** Performance metrics for metabolic syndrome classification by sex, mean and standard deviation

Sex	Features	AUC	AUPRC
Male	Age, Attenuation	0.601 (0.056)	0.334 (0.129)
	Age, IDP	0.785 (0.026)	0.543 (0.046)
	Age, VAT	0.770 (0.027)	0.532 (0.046)
	Age, VAT, IDP	0.810 (0.023)	0.587 (0.042)
Female	Age, Attenuation	0.664 (0.089)	0.241 (0.131)
	Age, IDP	0.812 (0.053)	0.419 (0.122)
	Age, VAT	0.819 (0.057)	0.391 (0.130)
	Age, VAT, IDP	0.848 (0.054)	0.460 (0.128)

*IDP* image-derived phenotype; *AUC* area under receiver-operating curve; *AUPRC* area under precision-recall curve; *Attenuation* median volumetric liver attenuation (expressed as Hounsfield units); *VAT* visceral adipose tissue

To investigate the biological signal captured by the IDP, we analyzed the underlying differences in etiology between sex. Among males, the most prevalent components for diagnosing metabolic syndrome were glucose levels, blood pressure, and waist circumference (Supplementary Fig. 1). In females, the most prevalent components were glucose levels, HDL levels, and waist circumference (Supplementary Fig. 1). Consistently, the IDP demonstrated the strongest correlations with waist circumference, glucose, and triglycerides for both males and females (Fig. 2b).

### IDP is associated with metabolic-related phenotypes

**Fig. 3:**
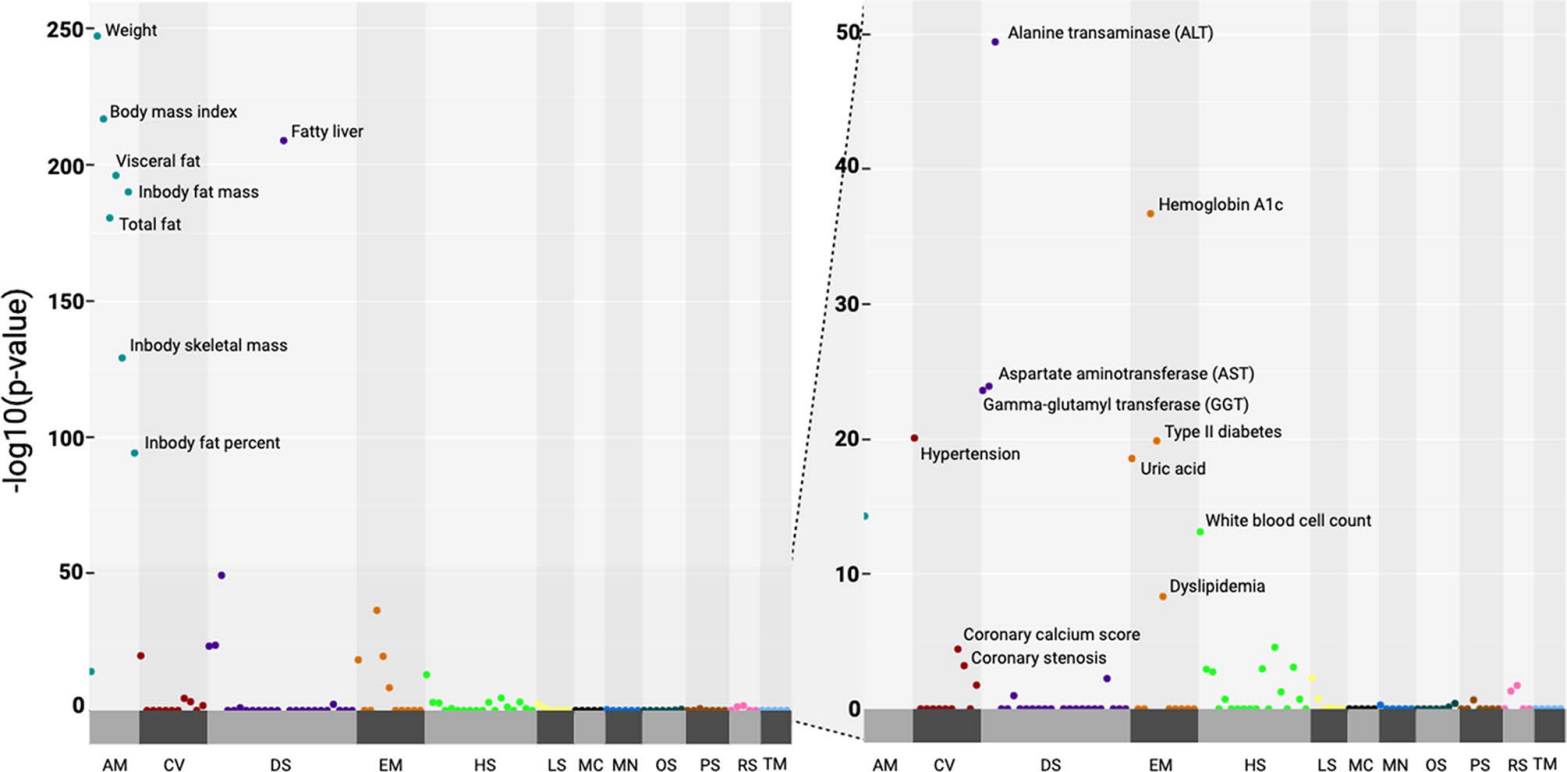
Phenotypic association analysis, corrected for age and sex. All *P* values are Bonferroni corrected. AM, anthropomorphic measurements; CV, cerebro-cardiovascular; DS, digestive system; EM, endocrine and metabolism; HS, hematologic system; LS, lifestyle; MC, musculoskeletal; OS, ophthalmic system; PS, pulmonary system; RS, renal system; TM, tumor markers

To explore the biological relevance of the IDP, we performed a phenome-wide association analysis (PheWAS) between 115 phenotypes corroborated by comprehensive health check-ups and the output of the IDP classifier (Supplementary Table 4). We found that the IDP is significantly associated with 32 phenotypes after Bonferroni correction (Fig. 3 and Supplementary Table 4). Notably, the most significant associations were observed with anthropometric measurements, including fat and muscle measurements, which are highly correlated with waist circumference, a component of metabolic syndrome. Other metabolic syndrome related phenotypes, including hypertension, hemoglobin A1c, type II diabetes diagnosis, and dyslipidemia, are also strongly associated. Hematologic features, such as white blood cell counts, as well as the endocrine phenotype uric acid are also significant. Additionally, digestive features related to the liver, such as alanine transaminase (ALT), aspartate transaminase (AST), and gamma-glutamyl transferase (GGT), demonstrated notable associations with the IDP. Of particular interest, the IDP exhibited a strong association with fatty liver disease compared to its association with liver attenuation (Supplementary Table 4). These findings suggest that the IDP, developed to classify metabolic syndrome, may also possess predictive capabilities for other metabolic-related conditions.

### IDP predicts future occurrence of metabolic-related morbidities

**Fig. 4:**
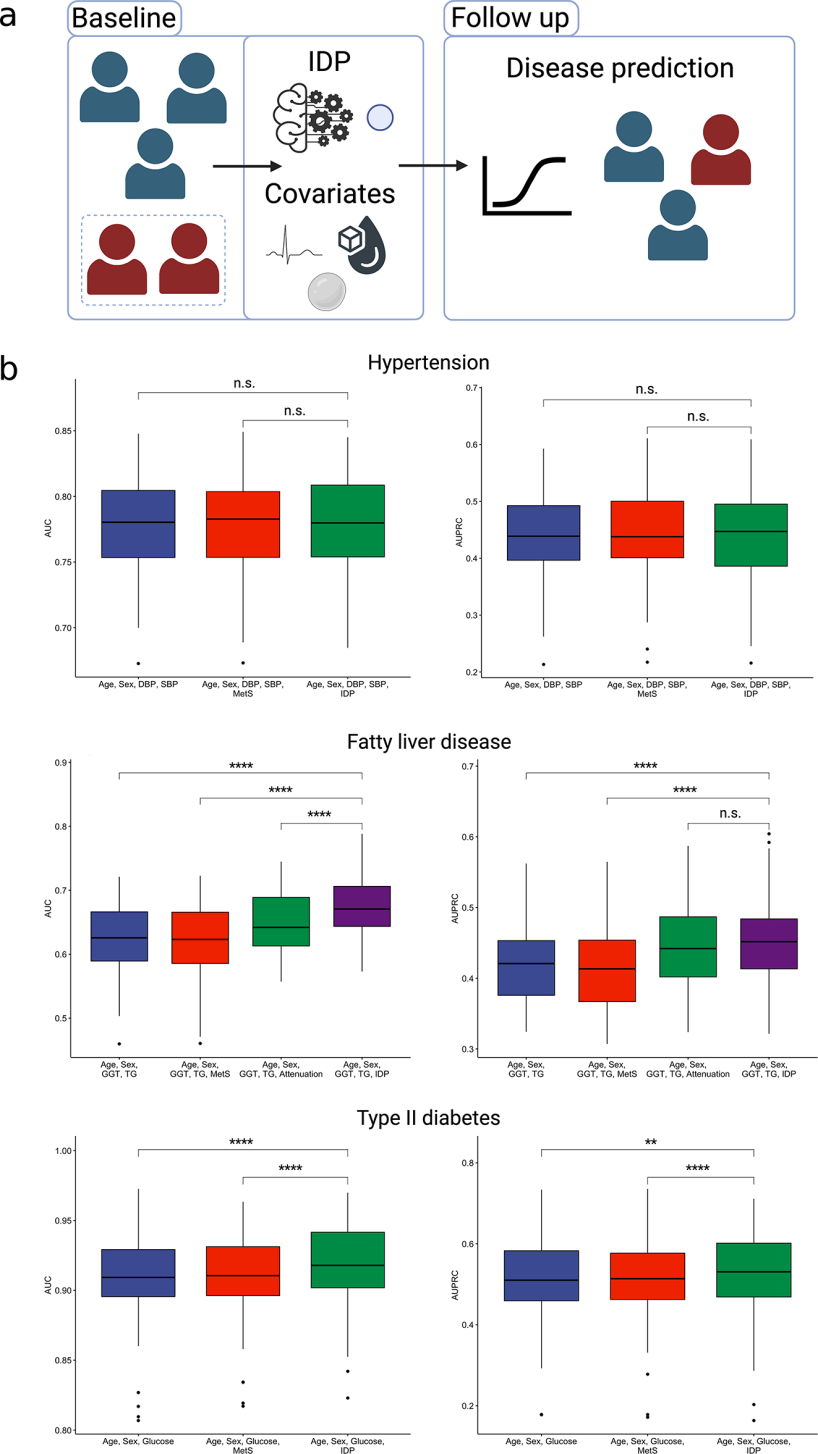
Overview of the follow-up disease prediction framework. Individuals diagnosed with the disease of interest as baseline are removed, and the baseline IDP and covariates are extracted from the remaining individuals. These are used as features to predict disease in the follow-up period. **b**, Predictive performance comparing relevant covariates and baseline metabolic syndrome diagnosis to the IDP. First row, hypertension; second row, type II diabetes; third row, fatty liver disease. SBP, systolic blood pressure; DBP, diastolic blood pressure; MetS, baseline metabolic syndrome; GGT, gamma-glutamyl transferase; TG, triglycerides; IDP, image-derived phenotype; AUC, area under receiver-operating curve.**** *P* < 0.0001, ** *P* < 0.001, n.s. not significant, paired t-test

We conducted predictive analyses for three morbidities—hypertension, fatty liver disease, and type II diabetes. Briefly, for each disease, we removed all individuals at baseline diagnosed with the respective condition, collected baseline IDP, covariates, and metabolic syndrome status from the remaining individuals, and predicted future disease (Fig. 4a). For these analyses, we consider the output of the classifier as a condensed representation of the IDP.

For hypertension prediction, we incorporated baseline blood pressure measurements as additional covariates. We found that the deep learning IDP did not improve the performance of predicting hypertension (Fig. 4b; Table 4). We included baseline GGT levels, TG levels, and liver attenuation as additional covariates for fatty liver disease prediction. Our analysis demonstrated that the IDP model significantly outperformed the model with covariates alone and the model including baseline clinical metabolic syndrome (Fig. 4b; Table 4). For type II diabetes prediction, baseline glucose levels were included. Once again, we observed that the IDP outperformed the two baseline models (Fig. 4b; Table 4). We see the same trends for disease prediction in models with only age and sex as covariates (Supplementary Table 5).

**Table 4:** Performance metrics for follow-up disease prediction (*n* = 1,397), mean and standard deviation

Disease	Features	AUC	AUPRC
Hypertension	Age, Sex, SBP, DBP	0.765 (0.039)	0.439 (0.082)
	Age, Sex, SBP, DBP, MetS	0.765 (0.040)	0.441 (0.081)
	Age, Sex, SBP, DBP, IDP	0.768 (0.038)	0.444 (0.080)
Fatty liver disease	Age, Sex, GGT, TG	0.625 (0.057)	0.422 (0.065)
	Age, Sex, GGT, TG, MetS	0.619 (0.062)	0.416 (0.065)
	Age, Sex, GGT, TG, Attenuation	0.647 (0.050)	0.449 (0.062)
	Age, Sex, GGT, TG, IDP	0.673 (0.046)	0.453 (0.065)
Type II diabetes	Age, Sex, Glucose	0.897 (0.041)	0.508 (0.110)
	Age, Sex, Glucose, MetS	0.903 (0.037)	0.508 (0.109)
	Age, Sex, Glucose, IDP	0.912 (0.035)	0.522 (0.108)

*AUC* area under receiver-operating curve; *AUPRC* area under precision-recall curve; *SBP* systolic blood pressure; *DBP* diastolic blood pressure; *MetS* baseline metabolic syndrome; *GGT* gamma-glutamyl transferase; *TG* triglycerides; *IDP* image-derived phenotype; *Attenuation* median volumetric liver attenuation (expressed as Hounsfield units)

We performed additional analyses in which individuals who were not diagnosed with metabolic syndrome at baseline were removed. We used logistic regression to compare the performance of models containing baseline covariates alone and models including the IDP. In all cases except for hypertension, the models including the IDP demonstrated superior performance compared to the covariate models (Supplementary Table 6).

To illustrate the robustness of the deep learning IDP, we identified cases where individuals appeared clinically healthy at baseline but subsequently developed metabolic-related morbidities in the follow-up period. Specifically, we selected individuals who had an IDP value that classified them as metabolically abnormal (Methods), however no diagnosis of metabolic syndrome or disease at baseline, a total of 705. We identified 34% (50/149) of the individuals who developed hypertension, 66% (126/190) of the individuals who developed fatty liver disease, and 78% (31/40) of the individuals who developed type II diabetes. Many of these individuals had two metabolic risk factors at baseline, high glucose levels being the most prevalent (Supplementary Fig. 4).

## Discussion

Metabolic syndrome represents a significant risk factor for the development of severe cardiometabolic diseases. It is characterized by a set of potentially reversible metabolic abnormalities, emphasizing the importance of early and accurate detection for clinical management. In this study, we successfully developed a deep learning-based image-derived phenotype of metabolic abnormality that can classify metabolic syndrome directly from unenhanced abdominal CT scans in a large cohort of over 2,000 individuals. Our approach involved training a 3D segmentation model to localize the liver and utilizing this region to predict metabolic syndrome through a combination of a pretrained segmentation encoder and a deep learning classifier. In comparison to traditional hand-crafted radiomic features that are known to be significantly associated with metabolic syndrome, volumetric liver attenuation and visceral adipose tissue, our analysis demonstrated that the deep learning IDP was able to more accurately classify metabolic syndrome than the radiomic features and clinical covariates alone.

We performed sex stratified analysis and found that for males, the IDP showed a stronger classification performance than visceral adipose tissue, while in females the opposite was shown, and visceral adipose tissue showed stronger performance. Studies suggest that visceral fat influences metabolic syndrome risk differently between sex, posing a greater risk for females. This risk is linked more to fat composition and distribution rather than total fat quantity [42].. Males tend to accumulate fat viscerally, predominantly in the abdomen, while females predominantly store it subcutaneously [43, 44]. Thus, the risk of metabolic syndrome is often assessed based on sex-specific visceral fat thresholds rather than absolute amounts [45]. This implies that females may be more metabolically sensitive to visceral fat accumulation.

The IDP was strongly correlated with each of the metabolic risk components and showed significant associations with 32 clinical phenotypes, many of which are directly related to cardiometabolic abnormalities. Given that the IDP is derived from imaging focused on the liver, liver-specific features including alanine transaminase (ALT), aspartate transaminase (AST), and gamma-glutamyl transferase (GGT), and fatty liver disease were some of the most strongly associated phenotypes.

To explore the clinical utility of the IDP, we further investigated its predictive capabilities for future occurrences of cardiometabolic diseases, including hypertension, fatty liver disease, and type II diabetes in a subset of over 1,300 individuals who had follow-up data. Inclusion of the IDP significantly improved predictive performance for each of the diseases when compared to baseline covariate models for fatty liver disease and type II diabetes. Our analyses clearly demonstrated that the IDP outperformed the clinical definition of metabolic syndrome in terms of predictive accuracy. We hypothesize that this may be due to the nature of the clinical metabolic syndrome definition. There are sixteen possible combinations of five risk factors for diagnosis, but an individual can have up to two risk factors and not be diagnosed. Due to the heterogeneity of the diagnosis, the proposed IDP may be learning information at a more granular scale. Additionally, the IDP successfully identified a subset of individuals who appeared clinically healthy at baseline but later developed a cardiometabolic disease during the follow-up period. Specifically, the IDP identified 34%, 66%, and 78% of the individuals who developed hypertension, fatty liver disease, and type II diabetes, respectively, despite these individuals not meeting the criteria for a metabolic syndrome diagnosis at baseline.

We validated the IDP in an external validation dataset, which was collected for a different purpose (colorectal cancer patients) compared to the comprehensive health check-up cohort. This difference in data collection may have contributed to the reduced performance observed in the external validation, as the underlying characteristics and disease profiles of the cohorts varied. Although the performance in classifying metabolic syndrome decreased, it remained significantly higher than the hand-crafted feature and covariate models. The reduced performance could also be attributed to inherent differences in data resulting from variations in devices and acquisition protocols used for obtaining the abdominal CT scans. Domain shift, which refers to a change in the underlying data distribution, is a well-known phenomenon in machine learning research that can lead to challenges in model translation from the training data to external datasets. This issue is particularly prevalent especially in medical imaging research, where datasets are collected within specific scopes that encompass factors such as time-frame, demography, clinical settings, and acquisition devices [46]. It is important to note that while the external validation cohort differed in its intended use, it still provided valuable insights into the generalizability and robustness of the IDP model. The variations observed between the cohorts emphasize the need for further validation in diverse populations and contexts to establish the broader applicability of the IDP.

This study has some limitations that need to be addressed in future research, including the characteristics of the selected cohort and the interpretability of the model. The comprehensive health check-up population consisted mainly of individuals who were generally healthy, which limited our ability to study a broader range of cardiometabolic diseases. We chose hypertension, fatty liver disease, and type II diabetes due to their moderate representation in the dataset. While we were able to validate the IDP for metabolic syndrome prediction using an external cohort, we were unable to externally validate the prediction of subsequent diseases in the follow-up analyses. Additionally, all individuals in this study belonged to a Korean population, necessitating replication in more diverse populations. Further, due to lack of comprehensive annotated data, the proposed IDP pipeline focused only on the liver. We would expect additional information would be found from other regions of the scan, as suggested by previous work [18]. Finally, while the deep learning IDP is a stronger representation of metabolic abnormality, it lacks biological interpretability. In future work, additional analyses need to be performed in order to better understand the underlying biological processes driving the performance of the proposed IDP and validating its connections with individual metabolic risk factors. Additionally, it is necessary to conduct prospective studies in diverse clinical settings to further validate our method and explore the utility of the IDP guiding personalized treatment strategies.

## Conclusions

This work establishes a robust framework for data-driven IDP discovery in the context of metabolic abnormality. It emphasizes the efficient approach of using non-specific CT scans for IDP extraction and underscores the utility of deep learning-driven imaging phenotypes as valuable tools in the assessment and management of metabolic syndrome and cardiometabolic disorders. Abdominal CT scans, performed for various reasons, currently provide insights restricted to the radiologist’s analysis, primarily concentrating on specific organ irregularities. However, providing the possibility of additional imaging phenotypes in patients undergoing abdominal CT for pathological reasons or routine check-up could provide additional valuable medical information for intervening before other complications arise, thus promoting overall health. While this work focuses on the liver region of unenhanced abdominal CT scans and cardiometabolic disorders, the general framework can be readily adapted for other diseases and regions of interest segmented from various imaging modalities.

### Electronic supplementary material

Below is the link to the electronic supplementary material.

Supplementary Material 1

## Data Availability

Code to reproduce the models described in this paper can be found at www.github.com/leibyj/MetabolicDisorders-IDP. The datasets analyzed during the current study are available from the corresponding author upon reasonable request.

## Declarations

**Ethics**.

The Institutional Review Board of Seoul National University Hospital approved the study protocol (H-1902-017-1008), and the study was conducted in accord with the Helsinki Declaration. Informed consent was waived by the board.

## Abbreviations

3D: Three-dimensional
ALT: Alanine transaminase
AST: Aspartate transaminase
AUC: Area under the receiver operating curve
AURPC: Area under the precision-recall curve
CT: Computed tomography
DICOM: Digital Imaging and Communications in Medicine
GGT: Gamma-glutamyl transferase
HDL: High-density lipoprotein
IDP: Image-derived phenotype
MetS: Metabolic syndrome
NIfTI: Neuroimaging Informatics Technology Initiative
TG: triglycerides
VAT: Visceral adipose tissue
