# Supplementary material for: Deep learning imaging phenotype can classify metabolic syndrome and is predictive of cardiometabolic disorders
Source: J Transl Med. 2024 May 8;22:434. doi: 10.1186/s12967-024-05163-1 (PMC11077781; doi:10.1186/s12967-024-05163-1)

Supplementary Table 1. Characteristics of the follow-up development cohort [29]. P-values are from t-test for continuous variables and the Fisher exact test for categorical variables.

|  | **Follow up metabolic syndrome (-)** | **Follow up metabolic syndrome (+)** | **P value** | **N** |
| --- | --- | --- | --- | --- |
| Baseline Age | 53.7 ± 8.4 | 54.1 ± 8.2 | 0.484 | 1397 |
| Year of baseline enrollment | |  | 0.274 | 1397 |
| 2014 | 878 (89.2%) | 361 (87.4%) |  |  |
| 2015 | 83 (8.4%) | 45 (10.9%) |  |  |
| 2016 | 23 (2.3%) | 7 (1.7%) |  |  |
| Gender |  |  | 0.015 | 1397 |
| Female | 321 (32.6%) | 107 (25.9%) |  |  |
| Male | 663 (67.4%) | 306 (74.1%) |  |  |
| Total fat amount (mm2) | 24769.2 ± 8161.9 | 33573.2 ± 9042.6 | < 0.001 | 1397 |
| Visceral fat amount (mm2) | 10561.1 ± 4910.2 | 15326.6 ± 5103.6 | < 0.001 | 1397 |
| Smoking (3 group) |  |  | < 0.001 | 1196 |
| None | 378 (44.7%) | 111 (31.7%) |  |  |
| Ex | 337 (39.8%) | 155 (44.3%) |  |  |
| Current | 131 (15.5%) | 84 (24.0%) |  |  |
| Smoking (2 group) |  |  | < 0.001 | 1196 |
| None | 378 (44.7%) | 111 (31.7%) |  |  |
| Ex, current | 468 (55.3%) | 239 (68.3%) |  |  |
| Alcohol consumption | |  | < 0.001 | 1203 |
| Social | 614 (72.2%) | 203 (57.5%) |  |  |
| Heavy | 236 (27.8%) | 150 (42.5%) |  |  |
| Hypertension diagnosis | |  | < 0.001 | 1397 |
| No | 839 (85.3%) | 251 (60.8%) |  |  |
| Yes | 145 (14.7%) | 162 (39.2%) |  |  |
| Hypertension medication | |  | < 0.001 | 1397 |
| No | 860 (87.4%) | 272 (65.9%) |  |  |
| Yes | 124 (12.6%) | 141 (34.1%) |  |  |
| Diabetes diagnosis |  |  | < 0.001 | 1397 |
| No | 939 (95.4%) | 366 (88.6%) |  |  |
| Yes | 45 (4.6%) | 47 (11.4%) |  |  |
| Diabetes medication | |  | < 0.001 | 1397 |
| No | 952 (96.7%) | 381 (92.3%) |  |  |
| Yes | 32 (3.3%) | 32 (7.7%) |  |  |
| Dyslipidemia diagnosis | |  | 0.002 | 1397 |
| No | 812 (82.5%) | 310 (75.1%) |  |  |
| Yes | 172 (17.5%) | 103 (24.9%) |  |  |
| Dyslipidemia medication | |  | 0.001 | 1397 |
| No | 878 (89.2%) | 341 (82.6%) |  |  |
| Yes | 106 (10.8%) | 72 (17.4%) |  |  |
| Systolic blood pressure | 114.5 ± 12.6 | 120.9 ± 12.7 | < 0.001 | 1395 |
| Diastolic blood pressure | 75.8 ±9.8 | 80.1 ±9.2 | < 0.001 | 1395 |
| Height | 167.0 ±7.4 | 168.5 ±7.9 | 0.001 | 1388 |
| Weight | 64.1 ± 10.2 | 72.9 ± 11.2 | < 0.001 | 1387 |
| Body mass index | 22.9 ±2.5 | 25.6 ±2.9 | < 0.001 | 1396 |
| In body skeletal muscle mass | 26.8 ±5.4 | 29.2 ±5.6 | < 0.001 | 1386 |
| In body fat mass | 15.7 ±4.2 | 20.6 ±5.4 | < 0.001 | 1386 |
| In body Fat percent | 24.7 ±5.8 | 28.3 ±5.8 | < 0.001 | 1386 |
| Waist circumference | 82.2 ±7.3 | 90.1 ±7.7 | < 0.001 | 1386 |
| Glucose | 97.1 ± 14.3 | 105.7 ± 19.1 | < 0.001 | 1380 |
| Triglycerides | 99.1 ± 56.0 | 144.4 ± 83.8 | < 0.001 | 1380 |
| HDL cholesterol | 55.1 ± 12.3 | 48.8 ± 10.8 | < 0.001 | 1380 |
| HBA1C | 5.6 ±0.5 | 5.9 ±0.6 | < 0.001 | 1386 |
| Metabolic risk: waist circumference | | | < 0.001 | 1386 |
| No | 808 (82.9%) | 179 (43.6%) |  |  |
| Yes | 167 (17.1%) | 232 (56.4%) |  |  |
| Metabolic risk: Triglycerides |  |  | < 0.001 | 1380 |
| No | 835 (85.7%) | 252 (62.1%) |  |  |
| Yes | 139 (14.3%) | 154 (37.9%) |  |  |
| Metabolic risk: HDL cholesterol | | | < 0.001 | 1380 |
| No | 871 (89.4%) | 289 (71.2%) |  |  |
| Yes | 103 (10.6%) | 117 (28.8%) |  |  |
| Metabolic risk: glucose | |  | < 0.001 | 1383 |
| No | 650 (66.9%) | 172 (41.8%) |  |  |
| Yes | 322 (33.1%) | 239 (58.2%) |  |  |
| Metabolic risk: hypertension | | | < 0.001 | 1397 |
| No | 685 (69.6%) | 171 (41.4%) |  |  |
| Yes | 299 (30.4%) | 242 (58.6%) |  |  |
| Baseline metabolic syndrome | |  | < 0.001 | 1397 |
| No | 891 (90.5%) | 216 (52.3%) |  |  |
| Yes | 93 (9.5%) | 197 (47.7%) |  |  |
| Renal stone | |  | 0.923 | 1396 |
| No | 950 (96.6%) | 398 (96.4%) |  |  |
| Yes | 33 (3.4%) | 15 (3.6%) |  |  |
| Liver hemangioma | |  | 0.564 | 1397 |
| No | 815 (82.8%) | 348 (84.3%) |  |  |
| Yes | 169 (17.2%) | 65 (15.7%) |  |  |
| Cholecystitis | |  | 0.766 | 1397 |
| No | 937 (95.2%) | 391 (94.7%) |  |  |
| Yes | 47 (4.8%) | 22 (5.3%) |  |  |
| Gallbladder stone | |  | 0.435 | 1397 |
| No | 923 (93.8%) | 382 (92.5%) |  |  |
| Yes | 61 (6.2%) | 31 (7.5%) |  |  |
| Gallbladder adenomyomatosis | |  | 0.202 | 1396 |
| No | 949 (96.5%) | 392 (94.9%) |  |  |
| Yes | 34 (3.5%) | 21 (5.1%) |  |  |
| Intraductal Papillary Mucinous Neoplasm |  |  | 0.789 | 1396 |
| No | 960 (97.7%) | 405 (98.1%) |  |  |
| Yes | 23 (2.3%) | 8 (1.9%) |  |  |
| Gallbladder polyp | |  | 0.789 | 1359 |
| No | 778 (81.3%) | 330 (82.1%) |  |  |
| Yes | 179 (18.7%) | 72 (17.9%) |  |  |
| Fatty liver |  |  | < 0.001 | 1393 |
| none | 643 (65.6%) | 133 (32.2%) |  |  |
| mild | 225 (23.0%) | 130 (31.5%) |  |  |
| moderate | 102 (10.4%) | 125 (30.3%) |  |  |
| severe | 10 (1.0%) | 25 (6.1%) |  |  |
| Cirrhosis |  |  | 0.886 | 1395 |
| No | 980 (99.8%) | 413 (100.0%) |  |  |
| Yes | 2 (0.2%) | 0 (0.0%) |  |  |
| Brain unidentified bright object | |  | 0.756 | 1025 |
| No | 474 (65.8%) | 197 (64.6%) |  |  |
| Yes | 246 (34.2%) | 108 (35.4%) |  |  |
| Brain small vessel disease | |  | 0.257 | 1025 |
| No | 647 (89.9%) | 266 (87.2%) |  |  |
| Yes | 73 (10.1%) | 39 (12.8%) |  |  |
| Brain atherosclerosis | |  | 0.967 | 1025 |
| No | 654 (90.8%) | 278 (91.1%) |  |  |
| Yes | 66 (9.2%) | 27 (8.9%) |  |  |
| Brain stenosis | |  | 1 | 1025 |
| No | 697 (96.8%) | 295 (96.7%) |  |  |
| Yes | 23 (3.2%) | 10 (3.3%) |  |  |
| Brain aneurysm | |  | 0.173 | 1025 |
| No | 691 (96.0%) | 286 (93.8%) |  |  |
| Yes | 29 (4.0%) | 19 (6.2%) |  |  |
| Brain atrophy | |  | 0.361 | 1025 |
| No | 705 (97.9%) | 295 (96.7%) |  |  |
| Yes | 15 (2.1%) | 10 (3.3%) |  |  |
| Breast cancer | |  | < 0.001 | 428 |
| No | 320 (99.7%) | 100 (93.5%) |  |  |
| Yes | 1 (0.3%) | 7 (6.5%) |  |  |
| Colorectal cancer | |  | 1 | 1397 |
| No | 982 (99.8%) | 412 (99.8%) |  |  |
| Yes | 2 (0.2%) | 1 (0.2%) |  |  |
| Coronary calcium scoring | 72.1 ± 238.7 | 110.0 ± 265.5 | 0.055 | 706 |
| Coronary stenosis | |  | 0.015 | 706 |
| No | 338 (72.7%) | 153 (63.5%) |  |  |
| Yes | 127 (27.3%) | 88 (36.5%) |  |  |
| Aorta dilatation | |  | 1 | 706 |
| No | 430 (92.5%) | 223 (92.5%) |  |  |
| Yes | 35 (7.5%) | 18 (7.5%) |  |  |
| Coronary plaque | |  | 0.057 | 706 |
| No | 266 (57.2%) | 119 (49.4%) |  |  |
| Yes | 199 (42.8%) | 122 (50.6%) |  |  |
| DEXA bone density | |  | < 0.001 | 973 |
| Normal | 493 (71.0%) | 230 (82.4%) |  |  |
| Osteopenia | 182 (26.2%) | 48 (17.2%) |  |  |
| Osteoporosis | 19 (2.7%) | 1 (0.4%) |  |  |
| Spine spondylosis | |  | 0.198 | 643 |
| No | 206 (45.3%) | 74 (39.4%) |  |  |
| Yes | 249 (54.7%) | 114 (60.6%) |  |  |
| Spine spondylolisthesis | |  | 0.512 | 643 |
| No | 442 (97.1%) | 185 (98.4%) |  |  |
| Yes | 13 (2.9%) | 3 (1.6%) |  |  |
| Spine compression fracture | |  | 0.632 | 643 |
| No | 446 (98.0%) | 186 (98.9%) |  |  |
| Yes | 9 (2.0%) | 2 (1.1%) |  |  |
| Spine disc narrowing | |  | 0.27 | 643 |
| No | 379 (83.3%) | 149 (79.3%) |  |  |
| Yes | 76 (16.7%) | 39 (20.7%) |  |  |
| EKG rate | 64.8 ±9.5 | 66.9 ± 10.3 | < 0.001 | 1225 |
| Cataract |  |  | 0.346 | 1320 |
| No | 852 (91.2%) | 345 (89.4%) |  |  |
| Yes | 82 (8.8%) | 41 (10.6%) |  |  |
| Drusen |  |  | 0.362 | 1320 |
| No | 902 (96.6%) | 368 (95.3%) |  |  |
| Yes | 32 (3.4%) | 18 (4.7%) |  |  |
| Macular change | |  | 0.348 | 1320 |
| No | 898 (96.1%) | 366 (94.8%) |  |  |
| Yes | 36 (3.9%) | 20 (5.2%) |  |  |
| Optic disc cupping | |  | 1 | 1320 |
| No | 903 (96.7%) | 373 (96.6%) |  |  |
| Yes | 31 (3.3%) | 13 (3.4%) |  |  |
| Optic fiber loss | |  | 0.049 | 1320 |
| No | 883 (94.5%) | 353 (91.5%) |  |  |
| Yes | 51 (5.5%) | 33 (8.5%) |  |  |
| Gastric cancer | |  | 0.618 | 1397 |
| No | 975 (99.1%) | 411 (99.5%) |  |  |
| Yes | 9 (0.9%) | 2 (0.5%) |  |  |
| Atrophic gastritis | |  | 0.254 | 1389 |
| No | 404 (41.3%) | 155 (37.8%) |  |  |
| Yes | 575 (58.7%) | 255 (62.2%) |  |  |
| Intestinal metaplasia | |  | 0.474 | 1389 |
| No | 722 (73.7%) | 294 (71.7%) |  |  |
| Yes | 257 (26.3%) | 116 (28.3%) |  |  |
| Duodenal ulcer | |  | 0.491 | 1389 |
| No | 936 (95.6%) | 396 (96.6%) |  |  |
| Yes | 43 (4.4%) | 14 (3.4%) |  |  |
| Gastric ulcer | |  | 0.605 | 1389 |
| No | 961 (98.2%) | 400 (97.6%) |  |  |
| Yes | 18 (1.8%) | 10 (2.4%) |  |  |
| GERD |  |  | < 0.001 | 1389 |
| No | 840 (85.8%) | 314 (76.6%) |  |  |
| Yes | 139 (14.2%) | 96 (23.4%) |  |  |
| White blood cell count | 5.1 ±1.4 | 5.6 ±1.5 | < 0.001 | 1389 |
| Platelet count | 229.2 ± 48.7 | 236.0 ± 54.2 | 0.027 | 1389 |
| Segmented neutrophils | 56.0 ±8.3 | 56.8 ±8.4 | 0.1 | 1389 |
| Lymphocyte | 33.4 ±7.5 | 32.7 ±7.5 | 0.089 | 1389 |
| Monocyte | 5.3 ±1.3 | 5.2 ±1.2 | 0.601 | 1389 |
| Eosinophil | 2.8 ±2.1 | 2.8 ±2.1 | 0.821 | 1389 |
| Basophil | 0.6 ±0.3 | 0.6 ±0.3 | 0.856 | 1389 |
| Red blood cell count | 4.7 ±0.4 | 4.9 ±0.4 | < 0.001 | 1389 |
| Hemoglobin | 14.5 ±1.4 | 14.9 ±1.3 | < 0.001 | 1389 |
| Calcium | 9.2 ±0.3 | 9.2 ±0.4 | 0.089 | 1381 |
| Phosphorus | 3.5 ±0.5 | 3.5 ±0.5 | 0.767 | 1381 |
| Blood urea nitrogen | 14.4 ±3.4 | 14.6 ±3.4 | 0.251 | 1381 |
| Uric acid | 5.5 ±1.3 | 6.1 ±1.4 | < 0.001 | 1381 |
| Creatinine | 0.8 ±0.2 | 0.9 ±0.2 | 0.137 | 1381 |
| Sodium | 141.1 ±2.0 | 141.0 ±2.1 | 0.3 | 1378 |
| Potassium | 4.3 ±0.3 | 4.3 ±0.4 | 0.905 | 1378 |
| Chloride | 104.4 ±2.2 | 104.4 ±2.2 | 0.502 | 1378 |
| TCO2 | 27.1 ±2.6 | 26.8 ±2.6 | 0.041 | 1378 |
| Glomerular filtration rate | 89.1 ± 14.4 | 88.9 ± 14.5 | 0.805 | 1381 |
| Free thyroxine (T4) | 1.3 ±0.2 | 1.3 ±0.2 | 0.861 | 1178 |
| Thyroid stimulating hormone | 1.7 ±1.0 | 1.6 ±1.2 | 0.317 | 1197 |
| CA125 | 7.0 ±9.8 | 6.3 ±8.5 | 0.589 | 394 |
| CA19 9 | 7.9 ± 16.7 | 7.6 ±7.5 | 0.686 | 1240 |
| Alpha fetoprotein (AFP) | 1.9 ±1.4 | 1.9 ±1.2 | 0.385 | 1243 |
| Carcinoembryonic antigen (CEA) | 1.2 ±0.6 | 1.2 ±0.6 | 0.486 | 1241 |
| Prostate-specific antigen (PSA) | 1.0 ±0.9 | 1.0 ±1.0 | 0.557 | 838 |
| Vitamin D3 | 20.1 ±7.4 | 20.4 ±6.7 | 0.596 | 997 |
| Total cholesterol | 194.2 ± 33.0 | 194.1 ± 34.6 | 0.955 | 1390 |
| Serum protein | 7.2 ±0.4 | 7.3 ±0.4 | 0.018 | 1381 |
| Serum albumin | 4.5 ±0.2 | 4.6 ±0.3 | < 0.001 | 1381 |
| Serum total bilrubin | 1.0 ±0.4 | 1.0 ±0.4 | 0.539 | 1381 |
| Alkaline phosphatase (ALP) | 53.1 ± 14.2 | 56.4 ± 15.0 | < 0.001 | 1381 |
| Glutamic oxaloacetic transaminase | 23.3 ±9.4 | 26.4 ± 12.4 | < 0.001 | 1380 |
| Glutamic pyruvic transaminase | 22.1 ± 14.2 | 29.9 ± 18.7 | < 0.001 | 1380 |
| Gamma-Glutamyl Transferase | 31.8 ± 28.7 | 49.9 ± 66.0 | < 0.001 | 1390 |
| LDL cholesterol | 122.2 ± 30.0 | 124.7 ± 31.1 | 0.162 | 1379 |
| Mean corpuscular volume | 92.2 ±4.0 | 91.8 ±3.8 | 0.084 | 1389 |
| Mean corpuscular hemoglobin | 30.5 ±1.7 | 30.6 ±1.5 | 0.078 | 1389 |
| Mean corpuscular hemoglobin concentration | 33.1 ±1.2 | 33.4 ±1.2 | < 0.001 | 1389 |
| Procalcitonin | 0.2 ±0.0 | 0.2 ±0.0 | 0.004 | 1389 |
| Mean platelet volume | 8.6 ±0.7 | 8.7 ±0.7 | 0.193 | 1389 |
| Prothrombin time (PT) | 1.0 ±0.1 | 1.0 ±0.1 | 0.276 | 720 |
| Activated partial thromboplastin time (aPTT) | 33.1 ±3.2 | 32.6 ±2.6 | 0.059 | 720 |
| Hematocrit | 43.7 ±3.8 | 44.7 ±3.7 | < 0.001 | 1389 |
| Red cell distribution width (RDW) | 12.8 ±0.6 | 12.8 ±0.5 | 0.772 | 1389 |
| Intraocular pressure right | 12.4 ±2.9 | 12.7 ±2.8 | 0.059 | 1281 |
| Intraocular pressure left | 12.7 ±2.9 | 13.1 ±3.0 | 0.061 | 1281 |
| Urine albumin | |  | 0.922 | 1372 |
| Negative | 960 (99.5%) | 404 (99.3%) |  |  |
| Positive | 5 (0.5%) | 3 (0.7%) |  |  |
| Hepatitis B |  |  | 0.496 | 1356 |
| No | 925 (97.2%) | 389 (96.3%) |  |  |
| Yes | 27 (2.8%) | 15 (3.7%) |  |  |
| Hepatitis C |  |  | 1 | 1370 |
| No | 955 (99.3%) | 405 (99.3%) |  |  |
| Yes | 7 (0.7%) | 3 (0.7%) |  |  |
| Forced vital capacity L | 3.6 ±0.8 | 3.6 ±0.8 | 0.489 | 1393 |
| Forced vital capacity percent | 92.6 ± 11.3 | 90.9 ± 11.2 | 0.009 | 1393 |
| Forced expiratory volume (FEV1) L | 2.9 ±0.6 | 2.9 ±0.6 | 0.466 | 1393 |
| Forced expiratory volume (FEV1) percent | 101.3 ± 13.7 | 99.9 ± 13.2 | 0.076 | 1392 |
| Forced expiratory volume (FEV1) forced vital capacity percent | 80.0 ±5.7 | 80.2 ±5.8 | 0.641 | 1393 |
| Pulmonary function test | |  | 0.229 | 1393 |
| Normal | 845 (86.2%) | 340 (82.3%) |  |  |
| Restrictive | 9 (0.9%) | 5 (1.2%) |  |  |
| Obstructive | 97 (9.9%) | 56 (13.6%) |  |  |
| Mixed | 29 (3.0%) | 12 (2.9%) |  |  |
| Follow up diabetes medication | |  | < 0.001 | 1397 |
| No | 914 (92.9%) | 333 (80.6%) |  |  |
| Yes | 70 (7.1%) | 80 (19.4%) |  |  |
| Follow up hypertension medication | |  | < 0.001 | 1397 |
| No | 765 (77.7%) | 178 (43.1%) |  |  |
| Yes | 219 (22.3%) | 235 (56.9%) |  |  |
| Follow up Dyslipidemia medication | |  | < 0.001 | 1397 |
| No | 633 (64.3%) | 196 (47.5%) |  |  |
| Yes | 351 (35.7%) | 217 (52.5%) |  |  |
| Follow up waist circumference | 83.6 ±6.8 | 92.4 ±6.9 | < 0.001 | 1397 |
| Follow up systolic blood pressure | 116.7 ± 12.6 | 123.7 ± 13.6 | < 0.001 | 1397 |
| Follow up diastolic blood pressure | 75.3 ±8.8 | 79.6 ±9.3 | < 0.001 | 1397 |
| Follow up height | 167.0 ±7.5 | 168.5 ±8.0 | 0.001 | 1397 |
| Follow up weight | 64.1 ± 10.0 | 73.9 ± 11.2 | < 0.001 | 1397 |
| Follow up fasting blood sugar | 99.9 ± 14.4 | 113.5 ± 19.1 | < 0.001 | 1396 |
| Follow up Triglycerides | 91.0 ± 47.9 | 146.2 ± 98.5 | < 0.001 | 1397 |
| Follow up HDL | 57.7 ± 13.1 | 47.9 ± 10.3 | < 0.001 | 1397 |
| Follow up HbA1c | 5.7 ±0.5 | 6.0 ±0.6 | < 0.001 | 1395 |
| Follow up metabolic risk: Wait circumference | |  | < 0.001 | 1397 |
| No | 720 (73.2%) | 50 (12.1%) |  |  |
| Yes | 264 (26.8%) | 363 (87.9%) |  |  |
| Follow up metabolic risk: Triglycerides | |  | < 0.001 | 1397 |
| No | 916 (93.1%) | 238 (57.6%) |  |  |
| Yes | 68 (6.9%) | 175 (42.4%) |  |  |
| Follow up metabolic risk: HDL cholesterol | |  | < 0.001 | 1397 |
| No | 935 (95.0%) | 269 (65.1%) |  |  |
| Yes | 49 (5.0%) | 144 (34.9%) |  |  |
| Follow up metabolic risk: Systolic blood pressure | |  | < 0.001 | 1397 |
| No | 845 (85.9%) | 268 (64.9%) |  |  |
| Yes | 139 (14.1%) | 145 (35.1%) |  |  |
| Follow up metabolic risk: Diastolic blood pressure | |  | < 0.001 | 1397 |
| No | 840 (85.4%) | 276 (66.8%) |  |  |
| Yes | 144 (14.6%) | 137 (33.2%) |  |  |
| Follow up metabolic risk: Hypertension | |  | < 0.001 | 1397 |
| No | 625 (63.5%) | 78 (18.9%) |  |  |
| Yes | 359 (36.5%) | 335 (81.1%) |  |  |
| Follow up metabolic risk: diabetes | |  | < 0.001 | 1397 |
| No | 580 (58.9%) | 46 (11.1%) |  |  |
| Yes | 404 (41.1%) | 367 (88.9%) |  |  |
| Follow up metabolic risk score count | 1.2 ±0.8 | 3.4 ±0.6 | < 0.001 | 1397 |

Supplementary Table 2. Extended characteristics of the baseline development cohort. P-values are from t-test for continuous variables and the Fisher exact test for categorical variables.

|  | **Metabolic syndrome (-)** | **Metabolic syndrome (+)** | **P value** | **N** |
| --- | --- | --- | --- | --- |
| Baseline Age | 53.4 ±8.4 | 55.0 ±8.0 | < 0.001 | 2268 |
| Year of baseline enrollment |  |  | 0.203 | 2268 |
| 2014 | 1607 (90.1%) | 430 (88.8%) |  |  |
| 2015 | 145 (8.1%) | 49 (10.1%) |  |  |
| 2016 | 32 (1.8%) | 5 (1.0%) |  |  |
| Gender |  |  | < 0.001 | 2268 |
| Female | 636 (35.7%) | 74 (15.3%) |  |  |
| Male | 1148 (64.3%) | 410 (84.7%) |  |  |
| Total fat amount (mm2) | 25185.2 ± 8438.1 | 34310.8 ± 8739.1 | < 0.001 | 2268 |
| Visceral fat amount (mm2) | 10577.3 ± 4873.6 | 16508.3 ± 4947.0 | < 0.001 | 2268 |
| Smoking (3 group) |  |  | < 0.001 | 1931 |
| None | 678 (44.9%) | 107 (25.5%) |  |  |
| Ex | 560 (37.1%) | 213 (50.7%) |  |  |
| Current | 273 (18.1%) | 100 (23.8%) |  |  |
| Smoking (2 group) |  |  | < 0.001 | 1931 |
| None | 678 (44.9%) | 107 (25.5%) |  |  |
| Ex, current | 833 (55.1%) | 313 (74.5%) |  |  |
| Alcohol consumption |  |  | < 0.001 | 1938 |
| Social | 1082 (71.9%) | 239 (55.2%) |  |  |
| Heavy | 423 (28.1%) | 194 (44.8%) |  |  |
| Hypertension diagnosis |  |  | < 0.001 | 2268 |
| No | 1527 (85.6%) | 245 (50.6%) |  |  |
| Yes | 257 (14.4%) | 239 (49.4%) |  |  |
| Hypertension medication |  |  | < 0.001 | 2268 |
| No | 1576 (88.3%) | 273 (56.4%) |  |  |
| Yes | 208 (11.7%) | 211 (43.6%) |  |  |
| Diabetes diagnosis |  |  | < 0.001 | 2268 |
| No | 1715 (96.1%) | 407 (84.1%) |  |  |
| Yes | 69 (3.9%) | 77 (15.9%) |  |  |
| Diabetes medication |  |  | < 0.001 | 2268 |
| No | 1738 (97.4%) | 431 (89.0%) |  |  |
| Yes | 46 (2.6%) | 53 (11.0%) |  |  |
| Dyslipidemia diagnosis |  |  | < 0.001 | 2268 |
| No | 1500 (84.1%) | 353 (72.9%) |  |  |
| Yes | 284 (15.9%) | 131 (27.1%) |  |  |
| Dyslipidemia medication |  |  | < 0.001 | 2268 |
| No | 1609 (90.2%) | 397 (82.0%) |  |  |
| Yes | 175 (9.8%) | 87 (18.0%) |  |  |
| Systolic blood pressure | 114.7 ± 12.5 | 123.4 ± 12.8 | < 0.001 | 2263 |
| Diastolic blood pressure | 75.7 ±9.6 | 82.5 ±9.2 | < 0.001 | 2263 |
| Height | 166.7 ±7.7 | 169.6 ±6.9 | < 0.001 | 2247 |
| Weight | 64.0 ± 10.2 | 75.4 ± 10.3 | < 0.001 | 2246 |
| Body mass index | 22.9 ±2.6 | 26.1 ±2.7 | < 0.001 | 2260 |
| In body skeletal muscle mass | 26.6 ±5.5 | 30.4 ±5.0 | < 0.001 | 2244 |
| In body fat mass | 16.0 ±4.5 | 21.1 ±5.2 | < 0.001 | 2244 |
| In body Fat percent | 25.1 ±6.2 | 28.0 ±5.4 | < 0.001 | 2244 |
| Waist circumference | 82.3 ±7.3 | 92.1 ±6.8 | < 0.001 | 2245 |
| Glucose | 95.9 ± 13.6 | 112.1 ± 19.7 | < 0.001 | 2246 |
| Triglycerides | 95.0 ± 53.4 | 176.8 ± 96.1 | < 0.001 | 2242 |
| HDL cholesterol | 54.8 ± 12.0 | 45.9 ±9.6 | < 0.001 | 2242 |
| HBA1C | 5.6 ±0.5 | 6.0 ±0.8 | < 0.001 | 2251 |
| Metabolic risk: waist circumference | |  | < 0.001 | 2245 |
| No | 1478 (83.9%) | 122 (25.3%) |  |  |
| Yes | 284 (16.1%) | 361 (74.7%) |  |  |
| Metabolic risk: Triglycerides |  |  | < 0.001 | 2242 |
| No | 1584 (90.1%) | 185 (38.3%) |  |  |
| Yes | 175 (9.9%) | 298 (61.7%) |  |  |
| Metabolic risk: HDL cholesterol | |  | < 0.001 | 2242 |
| No | 1569 (89.2%) | 289 (59.8%) |  |  |
| Yes | 190 (10.8%) | 194 (40.2%) |  |  |
| Metabolic risk: glucose | |  | < 0.001 | 2249 |
| No | 1273 (72.1%) | 81 (16.7%) |  |  |
| Yes | 492 (27.9%) | 403 (83.3%) |  |  |
| Metabolic risk: hypertension | |  | < 0.001 | 2268 |
| No | 1285 (72.0%) | 102 (21.1%) |  |  |
| Yes | 499 (28.0%) | 382 (78.9%) |  |  |
| Renal stone |  |  | 0.605 | 2266 |
| No | 1723 (96.7%) | 465 (96.1%) |  |  |
| Yes | 59 (3.3%) | 19 (3.9%) |  |  |
| Liver hemangioma |  |  | 0.441 | 2267 |
| No | 1516 (85.0%) | 404 (83.5%) |  |  |
| Yes | 267 (15.0%) | 80 (16.5%) |  |  |
| Cholecystitis |  |  | 0.328 | 2267 |
| No | 1708 (95.8%) | 458 (94.6%) |  |  |
| Yes | 75 (4.2%) | 26 (5.4%) |  |  |
| Gallbladder stone |  |  | 0.007 | 2267 |
| No | 1683 (94.4%) | 440 (90.9%) |  |  |
| Yes | 100 (5.6%) | 44 (9.1%) |  |  |
| Gallbladder adenomyomatosis | |  | 0.833 | 2266 |
| No | 1718 (96.4%) | 465 (96.1%) |  |  |
| Yes | 64 (3.6%) | 19 (3.9%) |  |  |
| Intraductal Papillary Mucinous Neoplasm |  |  | 0.63 | 2266 |
| No | 1740 (97.6%) | 475 (98.1%) |  |  |
| Yes | 42 (2.4%) | 9 (1.9%) |  |  |
| Gallbladder polyp |  |  | 0.124 | 2197 |
| No | 1437 (83.1%) | 373 (79.9%) |  |  |
| Yes | 293 (16.9%) | 94 (20.1%) |  |  |
| Fatty liver |  |  | < 0.001 | 2252 |
| none | 1129 (63.7%) | 144 (30.0%) |  |  |
| mild | 426 (24.0%) | 149 (31.0%) |  |  |
| moderate | 195 (11.0%) | 158 (32.9%) |  |  |
| severe | 22 (1.2%) | 29 (6.0%) |  |  |
| Cirrhosis |  |  | 1 | 2255 |
| No | 1772 (99.9%) | 480 (99.8%) |  |  |
| Yes | 2 (0.1%) | 1 (0.2%) |  |  |
| Brain unidentified bright object |  |  | 0.626 | 1428 |
| No | 734 (67.4%) | 223 (65.8%) |  |  |
| Yes | 355 (32.6%) | 116 (34.2%) |  |  |
| Brain small vessel disease |  |  | 0.272 | 1428 |
| No | 985 (90.4%) | 299 (88.2%) |  |  |
| Yes | 104 (9.6%) | 40 (11.8%) |  |  |
| Brain atherosclerosis |  |  | 0.004 | 1428 |
| No | 993 (91.2%) | 290 (85.5%) |  |  |
| Yes | 96 (8.8%) | 49 (14.5%) |  |  |
| Brain stenosis |  |  | 0.703 | 1428 |
| No | 1054 (96.8%) | 326 (96.2%) |  |  |
| Yes | 35 (3.2%) | 13 (3.8%) |  |  |
| Brain aneurysm |  |  | 1 | 1428 |
| No | 1031 (94.7%) | 321 (94.7%) |  |  |
| Yes | 58 (5.3%) | 18 (5.3%) |  |  |
| Brain atrophy |  |  | 0.143 | 1428 |
| No | 1070 (98.3%) | 328 (96.8%) |  |  |
| Yes | 19 (1.7%) | 11 (3.2%) |  |  |
| Breast cancer |  |  | 0.812 | 710 |
| No | 626 (98.4%) | 72 (97.3%) |  |  |
| Yes | 10 (1.6%) | 2 (2.7%) |  |  |
| Colorectal cancer |  |  | 0.536 | 2268 |
| No | 1779 (99.7%) | 484 (100.0%) |  |  |
| Yes | 5 (0.3%) | 0 (0.0%) |  |  |
| Coronary calcium scoring | 72.8 ± 255.2 | 137.0 ± 323.3 | 0.003 | 1003 |
| Coronary stenosis |  |  | < 0.001 | 1003 |
| No | 547 (75.0%) | 164 (59.9%) |  |  |
| Yes | 182 (25.0%) | 110 (40.1%) |  |  |
| Aorta dilatation |  |  | 0.752 | 1003 |
| No | 674 (92.5%) | 251 (91.6%) |  |  |
| Yes | 55 (7.5%) | 23 (8.4%) |  |  |
| Coronary plaque |  |  | < 0.001 | 1003 |
| No | 439 (60.2%) | 110 (40.1%) |  |  |
| Yes | 290 (39.8%) | 164 (59.9%) |  |  |
| DEXA bone density |  |  | 0.016 | 1431 |
| Normal | 833 (72.1%) | 221 (80.4%) |  |  |
| Osteopenia | 296 (25.6%) | 51 (18.5%) |  |  |
| Osteoporosis | 27 (2.3%) | 3 (1.1%) |  |  |
| Spine spondylosis |  |  | < 0.001 | 891 |
| No | 332 (46.6%) | 52 (29.2%) |  |  |
| Yes | 381 (53.4%) | 126 (70.8%) |  |  |
| Spine spondylolisthesis |  |  | 0.236 | 891 |
| No | 689 (96.6%) | 168 (94.4%) |  |  |
| Yes | 24 (3.4%) | 10 (5.6%) |  |  |
| Spine compression fracture | |  | 0.382 | 891 |
| No | 700 (98.2%) | 177 (99.4%) |  |  |
| Yes | 13 (1.8%) | 1 (0.6%) |  |  |
| Spine disc narrowing |  |  | 0.264 | 891 |
| No | 589 (82.6%) | 140 (78.7%) |  |  |
| Yes | 124 (17.4%) | 38 (21.3%) |  |  |
| EKG rate | 65.0 ±9.6 | 68.5 ± 10.2 | < 0.001 | 2006 |
| Cataract |  |  | 0.009 | 2134 |
| No | 1535 (91.8%) | 404 (87.6%) |  |  |
| Yes | 138 (8.2%) | 57 (12.4%) |  |  |
| Drusen |  |  | 0.243 | 2134 |
| No | 1615 (96.5%) | 439 (95.2%) |  |  |
| Yes | 58 (3.5%) | 22 (4.8%) |  |  |
| Macular change |  |  | 0.76 | 2134 |
| No | 1608 (96.1%) | 441 (95.7%) |  |  |
| Yes | 65 (3.9%) | 20 (4.3%) |  |  |
| Optic disc cupping |  |  | 0.987 | 2134 |
| No | 1616 (96.6%) | 446 (96.7%) |  |  |
| Yes | 57 (3.4%) | 15 (3.3%) |  |  |
| Optic fiber loss |  |  | 0.057 | 2134 |
| No | 1590 (95.0%) | 427 (92.6%) |  |  |
| Yes | 83 (5.0%) | 34 (7.4%) |  |  |
| Gastric cancer |  |  | 0.503 | 2268 |
| No | 1769 (99.2%) | 482 (99.6%) |  |  |
| Yes | 15 (0.8%) | 2 (0.4%) |  |  |
| Atrophic gastritis |  |  | 0.004 | 2239 |
| No | 773 (43.8%) | 173 (36.4%) |  |  |
| Yes | 991 (56.2%) | 302 (63.6%) |  |  |
| Intestinal metaplasia |  |  | 0.001 | 2239 |
| No | 1348 (76.4%) | 328 (69.1%) |  |  |
| Yes | 416 (23.6%) | 147 (30.9%) |  |  |
| Duodenal ulcer |  |  | 0.931 | 2239 |
| No | 1682 (95.4%) | 454 (95.6%) |  |  |
| Yes | 82 (4.6%) | 21 (4.4%) |  |  |
| Gastric ulcer |  |  | 1 | 2239 |
| No | 1727 (97.9%) | 465 (97.9%) |  |  |
| Yes | 37 (2.1%) | 10 (2.1%) |  |  |
| GERD |  |  | < 0.001 | 2239 |
| No | 1518 (86.1%) | 365 (76.8%) |  |  |
| Yes | 246 (13.9%) | 110 (23.2%) |  |  |
| White blood cell count | 5.2 ±1.5 | 5.9 ±1.6 | < 0.001 | 2254 |
| Platelet count | 231.9 ± 52.9 | 233.4 ± 54.5 | 0.58 | 2254 |
| Segmented neutrophils | 56.7 ±8.5 | 56.7 ±8.1 | 0.905 | 2254 |
| Lymphocyte | 32.9 ±7.7 | 32.5 ±7.1 | 0.259 | 2254 |
| Monocyte | 5.2 ±1.3 | 5.3 ±1.1 | 0.376 | 2254 |
| Eosinophil | 2.7 ±2.0 | 3.1 ±2.1 | < 0.001 | 2254 |
| Basophil | 0.5 ±0.3 | 0.6 ±0.3 | 0.021 | 2254 |
| Red blood cell count | 4.8 ±0.4 | 5.0 ±0.4 | < 0.001 | 2254 |
| Hemoglobin | 14.5 ±1.4 | 15.1 ±1.3 | < 0.001 | 2254 |
| Calcium | 9.2 ±0.3 | 9.3 ±0.4 | 0.002 | 2246 |
| Phosphorus | 3.5 ±0.5 | 3.5 ±0.5 | 0.085 | 2246 |
| Blood urea nitrogen | 14.4 ±3.4 | 14.7 ±3.5 | 0.105 | 2246 |
| Uric acid | 5.5 ±1.3 | 6.3 ±1.4 | < 0.001 | 2246 |
| Creatinine | 0.8 ±0.2 | 0.9 ±0.2 | < 0.001 | 2246 |
| Sodium | 141.1 ±2.0 | 141.0 ±2.0 | 0.122 | 2231 |
| Potassium | 4.3 ±0.3 | 4.3 ±0.3 | 0.264 | 2231 |
| Chloride | 104.6 ±2.3 | 104.1 ±2.3 | < 0.001 | 2231 |
| TCO2 | 26.9 ±2.7 | 26.9 ±2.5 | 0.765 | 2231 |
| Glomerular filtration rate | 89.3 ± 14.2 | 88.9 ± 15.1 | 0.641 | 2246 |
| Free thyroxine (T4) | 1.3 ±0.2 | 1.3 ±0.3 | 0.176 | 1938 |
| Thyroid stimulating hormone | 1.6 ±1.1 | 1.7 ±1.3 | 0.7 | 1980 |
| CA125 | 8.0 ± 23.6 | 5.9 ±8.2 | 0.146 | 651 |
| CA19 9 | 7.7 ± 14.1 | 7.4 ±5.8 | 0.45 | 2036 |
| Alpha fetoprotein (AFP) | 1.9 ±1.4 | 1.9 ±1.5 | 0.545 | 2043 |
| Carcinoembryonic antigen (CEA) | 1.2 ±0.7 | 1.3 ±0.8 | 0.029 | 2040 |
| Prostate-specific antigen (PSA) | 1.0 ±0.9 | 1.0 ±0.8 | 0.435 | 1373 |
| Vitamin D3 | 20.0 ±7.2 | 20.0 ±6.8 | 0.999 | 1628 |
| Total cholesterol | 194.9 ± 33.4 | 191.8 ± 34.9 | 0.068 | 2260 |
| Serum protein | 7.3 ±0.4 | 7.3 ±0.4 | 0.008 | 2246 |
| Serum albumin | 4.5 ±0.2 | 4.6 ±0.3 | < 0.001 | 2246 |
| Serum total bilirubin | 1.0 ±0.4 | 1.0 ±0.4 | 0.409 | 2246 |
| Alkaline phosphatase (ALP) | 54.4 ± 14.3 | 55.7 ± 14.9 | 0.081 | 2246 |
| Glutamic oxaloacetic transaminase | 23.6 ±9.8 | 27.6 ± 13.5 | < 0.001 | 2245 |
| Glutamic pyruvic transaminase | 22.9 ± 15.3 | 32.0 ± 20.1 | < 0.001 | 2245 |
| Gamma-Glutamyl Transferase | 33.0 ± 32.3 | 55.9 ± 66.7 | < 0.001 | 2257 |
| LDL cholesterol | 123.9 ± 30.6 | 121.3 ± 30.3 | 0.099 | 2241 |
| Mean corpuscular volume | 92.1 ±4.1 | 91.2 ±4.1 | < 0.001 | 2254 |
| Mean corpuscular hemoglobin | 30.4 ±1.7 | 30.5 ±1.8 | 0.522 | 2254 |
| Mean corpuscular hemoglobin concentration | 33.1 ±1.2 | 33.4 ±1.2 | < 0.001 | 2254 |
| Procalcitonin | 0.2 ±0.0 | 0.2 ±0.0 | 0.811 | 2254 |
| Mean platelet volume | 8.6 ±0.8 | 8.6 ±0.7 | 0.816 | 2254 |
| Prothrombin time (PT) | 1.0 ±0.1 | 1.0 ±0.1 | 0.585 | 1235 |
| Activated partial thromboplastin time (aPTT ) | 33.1 ±3.0 | 32.5 ±3.0 | 0.007 | 1235 |
| Hematocrit | 43.8 ±3.9 | 45.3 ±3.7 | < 0.001 | 2254 |
| Red cell distribution width (RDW) | 12.9 ±0.7 | 12.9 ±0.6 | 0.644 | 2254 |
| Intraocular pressure right | 12.3 ±2.9 | 12.9 ±3.0 | < 0.001 | 2088 |
| Intraocular pressure left | 12.7 ±2.9 | 13.3 ±2.9 | < 0.001 | 2087 |
| Urine PH | 5.6 ±0.9 | 5.6 ±0.9 | 0.957 | 2230 |
| Urine albumin |  |  | 0.128 | 2230 |
| Negative | 1741 (99.4%) | 471 (98.5%) |  |  |
| Positive | 11 (0.6%) | 7 (1.5%) |  |  |
| Hepatitis B |  |  | 1 | 2194 |
| No | 1668 (96.5%) | 450 (96.6%) |  |  |
| Yes | 60 (3.5%) | 16 (3.4%) |  |  |
| Hepatitis C |  |  | 0.022 | 2219 |
| No | 1736 (99.5%) | 466 (98.3%) |  |  |
| Yes | 9 (0.5%) | 8 (1.7%) |  |  |
| Forced vital capacity L | 3.6 ±0.8 | 3.8 ±0.7 | < 0.001 | 2249 |
| Forced vital capacity percent | 93.0 ± 11.3 | 90.8 ± 10.7 | < 0.001 | 2249 |
| Forced expiratory volume (FEV1) L | 2.9 ±0.7 | 3.0 ±0.6 | 0.002 | 2249 |
| Forced expiratory volume (FEV1) percent | 101.3 ± 13.5 | 99.2 ± 13.1 | 0.003 | 2248 |
| Forced expiratory volume (FEV1) forced vital capacity percent | 80.1 ±6.0 | 79.4 ±6.0 | 0.028 | 2249 |
| Pulmonary function test |  |  | 0.302 | 2249 |
| Normal | 1540 (86.9%) | 398 (83.6%) |  |  |
| Restrictive | 19 (1.1%) | 7 (1.5%) |  |  |
| Obstructive | 163 (9.2%) | 52 (10.9%) |  |  |
| Mixed | 51 (2.9%) | 19 (4.0%) |  |  |

Supplementary Table 3. Characteristics of the external validation cohort [35].

|  | **N** | **Values** |
| --- | --- | --- |
| Gender | 521 |  |
| Male |  | 321 (61.6%) |
| Female |  | 200 (38.4%) |
| Age of operation | 521 | 61.6 ± 9.0 |
| Alcohol consumption | 521 |  |
| Social |  | 345 (66.2%) |
| Heavy |  | 176 (33.8%) |
| Smoking | 521 |  |
| None |  | 427 (82.0%) |
| Ex |  | 11 (2.1%) |
| Current |  | 83 (15.9%) |
| Diabetes medication | 521 |  |
| No |  | 450(86.4%) |
| Yes |  | 71(13.6%) |
| Hypertension medication | 521 |  |
| No |  | 313 (60.1%) |
| Yes |  | 208 (39.9%) |
| Dyslipidemia medication | 521 |  |
| No |  | 487 (93.5%) |
| Yes |  | 34 (6.5%) |
| Height | 521 | 162.4 ± 8.3 |
| Weight | 521 | 63.9 ± 10.6 |
| Systolic blood pressure | 521 | 128.0 ± 13.7 |
| Diastolic blood pressure | 520 | 79.9 ± 10.2 |
| Glucose | 513 | 110.3 ± 31.7 |
| Uric acid | 520 | 5.0 ± 1.4 |
| Blood urea nitrogen | 521 | 13.6 ± 4.7 |
| Creatinine | 521 | 0.9 ± 0.3 |
| Total cholesterol | 521 | 180.0 ± 33.9 |
| Albumin | 521 | 4.2 ± 0.4 |
| Glutamic oxaloacetic transaminase | 521 | 23.2 ± 9.3 |
| Glutamic pyruvic transaminase | 521 | 21.8 ± 12.1 |
| Total fat amount (mm2) | 520 | 26966.4 ± 9437.4 |
| Visceral fat amount (mm2) | 520 | 12216.3 ± 5416.9 |

Supplementary Table 4. Phenotype correlation analysis, corrected for age and sex. All p-values are Bonferroni corrected. The IDP and liver attenuation features are performed as independent models. IDP, image-derived phenotype; Attenuation; median volumetric liver attenuation (expressed as Hounsfield units).

| **N** | **Phenotype** | **IDP beta** | **IDP p-value** | **Liver attenuation beta** | **Liver attenuation p-value** |
| --- | --- | --- | --- | --- | --- |
| 2257 | Height | 0.24608242 | 5.22E-15 | 0.02264 | 1 |
| 2256 | White blood cell count | 0.77399095 | 6.79E-248 | -0.3653 | 2.42E-38 |
| 2271 | Body mass index | 0.59401858 | 1.85E-217 | -0.3411 | 4.56E-52 |
| 2266 | Weight | 0.15560281 | 7.64E-14 | -0.1997 | 1.62E-19 |
| 2266 | Red blood cell count | 0.10077667 | 0.00114013 | -0.209 | 3.02E-15 |
| 2266 | Hemoglobin | 0.1085051 | 0.001797069 | -0.1438 | 1.64E-05 |
| 2266 | Hematocrit | 0.04204251 | 1 | -0.1114 | 0.00194 |
| 2266 | Platelet count | 0.06201002 | 0.192054073 | -0.0928 | 0.00174 |
| 2268 | Gamma-Glutamyl Transferase | 0.20761085 | 2.45E-24 | -0.1236 | 9.22E-07 |
| 2255 | Aspartate Aminotransferase (AST) | 0.20427296 | 1.20E-24 | -0.3031 | 3.96E-47 |
| 2255 | Alanine transaminase (ALT) | 0.29027525 | 3.56E-50 | -0.3783 | 1.18E-73 |
| 2255 | Alkaline phosphatase (ALP) | 0.02024116 | 1 | -0.0945 | 0.00096 |
| 2255 | Albumin | 0.04739037 | 1 | -0.1342 | 2.84E-08 |
| 2255 | Blood urea nitrogen | 0.00529885 | 1 | -0.0615 | 0.56569 |
| 2255 | Creatinine | -0.095542 | 0.047777158 | 0.0081 | 1 |
| 2255 | Uric acid | 0.21246029 | 2.78E-19 | -0.2037 | 9.84E-15 |
| 2268 | Total cholesterol | 0.04814774 | 1 | -0.0673 | 0.14936 |
| 2249 | LDL cholesterol | 0.04987418 | 1 | -0.0919 | 0.00118 |
| 2255 | Calcium | 0.05459641 | 0.519936721 | -0.083 | 0.00844 |
| 2255 | Total bilirubin | -0.0665297 | 0.104136645 | -0.012 | 1 |
| 2259 | HbA1c | 0.25314414 | 1.86E-37 | -0.2756 | 3.80E-38 |
| 2255 | CKD-EPI | 0.07477278 | 0.018230687 | -0.0172 | 1 |
| 2279 | Total fat CT mm2 | 0.51014578 | 2.71E-181 | -0.3321 | 3.71E-58 |
| 2279 | Visceral fat CT mm2 | 0.59098249 | 9.69E-197 | -0.3988 | 9.62E-68 |
| 1955 | Smoking (3 group) | 0.10126302 | 0.005082766 | -0.0534 | 1 |
| 1955 | Smoking (2 group) | 0.08415637 | 0.174185235 | -0.0447 | 1 |
| 1948 | Alcohol heavy (> 14) | 0.05394339 | 1 | 0.0772 | 0.11609 |
| 1948 | Alcohol amount | 0.06119467 | 0.786103092 | 0.08178 | 0.05796 |
| 2280 | Hypertension diagnosis | 0.1936232 | 8.43E-21 | -0.1565 | 2.82E-11 |
| 2280 | Diabetes diagnosis | 0.18787427 | 1.36E-20 | -0.1422 | 1.12E-09 |
| 2280 | Dyslipid diagnosis | 0.12932295 | 4.55E-09 | -0.074 | 0.05723 |
| 2253 | In body skeletal muscle mass | 0.82372143 | 6.46E-130 | -0.234 | 1.67E-07 |
| 2253 | In body fat mass | 0.52331515 | 1.11E-190 | -0.3341 | 3.42E-58 |
| 2253 | In body Fat percent | 0.46770066 | 5.00E-95 | -0.3507 | 3.17E-43 |
| 2262 | Exercise continuous | 0.03004516 | 1 | 0.00311 | 1 |
| 2280 | Exercise categoric | 0.00203912 | 1 | 0.04662 | 1 |
| 2280 | Renal stone | -0.0012484 | 1 | -0.0175 | 1 |
| 2280 | Liver hemangioma | 0.0141359 | 1 | -0.0507 | 1 |
| 2280 | Cholecystitis | -0.0005565 | 1 | 0.00588 | 1 |
| 2280 | Gallbladder stone | 0.00766008 | 1 | -0.0238 | 1 |
| 2280 | Gallbladder adenomyomatosis | -0.0180607 | 1 | 0.02918 | 1 |
| 2280 | Intraductal Papillary Mucinous Neoplasm | 0.00826532 | 1 | 0.02356 | 1 |
| 2219 | Gallbladder polyp | 0.0256713 | 1 | 0.02587 | 1 |
| 2266 | Fatty liver | 0.55533826 | 1.63E-209 | -0.5954 | 2.54E-204 |
| 2269 | Cirrhosis | 0.00850869 | 1 | 0.00729 | 1 |
| 1451 | Brain unidentified bright object | 0.01779107 | 1 | 0.01758 | 1 |
| 1451 | Brain small vessel disease | 0.00238917 | 1 | -0.0144 | 1 |
| 1451 | Brain atherosclerosis | 0.00172082 | 1 | 0.00372 | 1 |
| 1451 | Brain stenosis | 0.00663364 | 1 | -0.0179 | 1 |
| 1451 | Brain aneurysm | -0.01922 | 1 | 0.01267 | 1 |
| 1451 | Brain atrophy | 0.02393591 | 1 | -0.0431 | 1 |
| 689 | Breast cancer | 0.06184563 | 1 | -0.1493 | 0.01677 |
| 2280 | Colorectal cancer | -0.0230568 | 1 | 0.01273 | 1 |
| 1038 | Coronary calcium score | 0.14898845 | 3.69E-05 | -0.0359 | 1 |
| 1038 | Coronary stenosis | 0.13346166 | 0.00061808 | -0.0673 | 1 |
| 1038 | Aorta dilatation | -0.0123553 | 1 | 0.0236 | 1 |
| 1038 | Coronary plaque | 0.11296871 | 0.017267354 | -0.0702 | 1 |
| 1415 | DEXA bone density | -0.0541542 | 1 | -0.0012 | 1 |
| 904 | Spine spondylosis | 0.01418781 | 1 | 0.03573 | 1 |
| 904 | Spine spondylolisthesis | -0.0235437 | 1 | 0.00612 | 1 |
| 904 | Spine compression fracture | -0.0156786 | 1 | -0.0046 | 1 |
| 904 | Spine disc narrowing | 0.01630796 | 1 | 0.0043 | 1 |
| 2133 | Cataract | -0.0072053 | 1 | -0.011 | 1 |
| 2133 | Drusen | 0.01587353 | 1 | -0.0437 | 1 |
| 2133 | Macular change | -0.009741 | 1 | 0.01527 | 1 |
| 2133 | Optic disc cupping | -0.0356312 | 1 | 0.01361 | 1 |
| 2133 | Optic fiber loss | 0.01029685 | 1 | -0.0004 | 1 |
| 2280 | Gastric cancer | 0.01173214 | 1 | -0.006 | 1 |
| 2255 | Atrophic gastritis | 0.01033661 | 1 | -0.0595 | 0.81928 |
| 2255 | Intestinal metaplasia | 0.01577361 | 1 | -0.0292 | 1 |
| 2255 | Duodenal ulcer | -0.0497641 | 1 | 0.02692 | 1 |
| 2255 | Gastric ulcer | 0.01252523 | 1 | -0.0007 | 1 |
| 2255 | GERD | 0.07839685 | 0.005591089 | -0.014 | 1 |
| 2266 | Segmented neutrophils | -0.0055769 | 1 | -0.0784 | 0.02021 |
| 2266 | Lymphocyte | -0.0009 | 1 | 0.06888 | 0.11398 |
| 2266 | Monocyte | -0.003279 | 1 | 0.0533 | 1 |
| 2266 | Eosinophil | 0.02666161 | 1 | 0.02778 | 1 |
| 2266 | Basophil | 0.04826714 | 1 | -0.0006 | 1 |
| 2255 | Phosphorus | 0.04297392 | 1 | -0.0486 | 1 |
| 2240 | sodium | -0.0507989 | 1 | 0.03123 | 1 |
| 2240 | Potassium | -0.0401411 | 1 | 0.04498 | 1 |
| 2240 | Chloride | -0.0280556 | 1 | 0.01001 | 1 |
| 2240 | TCO2 | -0.0456252 | 1 | 0.13763 | 6.01E-09 |
| 1934 | Free thyroxine (T4) | -0.043572 | 1 | 0.02054 | 1 |
| 1979 | Thyroid stimulating hormone | -0.0036902 | 1 | 0.02988 | 1 |
| 628 | CA125 | -0.0159496 | 1 | 0.00056 | 1 |
| 2043 | CA19 9 | -0.0016098 | 1 | -0.0099 | 1 |
| 2045 | Alpha fetoprotein (AFP) | -0.0490263 | 1 | 0.04197 | 1 |
| 2047 | Carcinoembryonic antigen (CEA) | -0.015058 | 1 | 0.02181 | 1 |
| 1396 | Prostate-specific antigen (PSA) | -0.0222766 | 1 | 0.00273 | 1 |
| 1606 | Vitamin D3 | -0.0333397 | 1 | 0.05371 | 1 |
| 2255 | Serum protein | 0.02555055 | 1 | -0.1277 | 1.07E-07 |
| 2266 | Mean corpuscular volume | -0.0871729 | 0.001057025 | 0.15973 | 7.34E-12 |
| 2266 | Mean corpuscular hemoglobin | -0.0040015 | 1 | 0.10018 | 0.00042 |
| 2266 | Mean corpuscular hemoglobin concentration | 0.10237353 | 2.65E-05 | -0.0436 | 1 |
| 2266 | Plateletcrit | 0.0694093 | 0.054724878 | -0.0693 | 0.15477 |
| 2266 | Mean platelet volume | 0.01922101 | 1 | 0.06362 | 0.27024 |
| 1262 | Prothrombin time (PT) | -0.1215243 | 0.000802502 | 0.00533 | 1 |
| 1262 | Activated partial thromboplastin time (aPTT) | -0.0852776 | 0.192119033 | 0.08262 | 0.49632 |
| 2266 | Red cell distribution width (RDW) | 0.01199019 | 1 | -0.0466 | 1 |
| 2084 | Intraocular pressure right | 0.05533867 | 0.724559585 | -0.0879 | 0.00847 |
| 2081 | Intraocular pressure left | 0.05945018 | 0.393332729 | -0.0984 | 0.00111 |
| 2244 | Urine albumin | 0.01846963 | 1 | -0.0166 | 1 |
| 2198 | Hepatitis B | -0.0213285 | 1 | -0.0551 | 1 |
| 2225 | Hepatitis C | -0.0043656 | 1 | 0.05321 | 1 |
| 2280 | Lung cancer | -0.0239947 | 1 | 0.01671 | 1 |
| 2260 | Forced vital capacity L | 0.04349894 | 1 | 0.13809 | 0.00286 |
| 2260 | Forced vital capacity percent | -0.0600417 | 0.216101937 | 0.09836 | 0.00029 |
| 2260 | Forced expiratory volume (FEV1) L | 0.06399987 | 1 | 0.12337 | 0.01482 |
| 2260 | Forced expiratory volume (FEV1) percent | -0.0310587 | 1 | 0.08659 | 0.00414 |
| 2260 | Forced expiratory volume (FEV1) forced vital capacity percent | 0.03507642 | 1 | -0.0122 | 1 |
| 2260 | Pulmonary function test | 0.00508021 | 1 | -0.0315 | 1 |
| 1591 | Prostate cancer | -0.0050079 | 1 | -0.0155 | 1 |
| 2280 | Thyroid cancer | 0.00801945 | 1 | -0.0321 | 1 |

Supplementary Table 5. Performance metrics for follow-up disease prediction (n=1,397), mean and standard deviation.

| **Disease** | **Features** | **AUC** | **AUPRC** |
| --- | --- | --- | --- |
| Hypertension | Age, Sex | 0.643 (0.048) | 0.278 (0.058) |
|  | Age, Sex, MetS | 0.672 (0.049) | 0.322 (0.082) |
|  | Age, Sex, IDP | 0.677 (0.044) | 0.296 (0.057) |
| Fatty liver disease | Age, Sex | 0.577 (0.047) | 0.370 (0.064) |
|  | Age, Sex, MetS | 0.589 (0.054) | 0.381 (0.061) |
|  | Age, Sex, Attenuation | 0.597 (0.046) | 0.403 (0.059) |
|  | Age, Sex, IDP | 0.654 (0.050) | 0.443 (0.068) |
| Type II diabetes | Age, Sex | 0.604 (0.056) | 0.088 (0.024) |
|  | Age, Sex, MetS | 0.739 (0.059) | 0.143 (0.050) |
|  | Age, Sex, IDP | 0.754 (0.055) | 0.164 (0.052) |

Supplementary Table 6. Performance metrics for follow-up disease prediction for individuals without baseline metabolic syndrome diagnosis (n=1,107), mean and standard deviation. SBP, systolic blood pressure; DBP, diastolic blood pressure; GGT, gamma-glutamyl transferase; TG, triglycerides; IDP, image-derived phenotype; AUC, area under receiver-operating curve; AUPRC, area under precision-recall curve; Attenuation, median volumetric liver attenuation (expressed as Hounsfield units); VAT, visceral adipose tissue.

| **Disease** | **Features** | **AUC** | **AUPRC** |
| --- | --- | --- | --- |
| Hypertension | Age, Sex, SBP, DBP | 0.756 (0.043) | 0.361 (0.078) |
|  | Age, Sex, SBP, DBP, IDP | 0.754 (0.043) | 0.360 (0.081) |
| Fatty liver | Age, Sex, GGT, TG | 0.624 (0.072) | 0.415 (0.065) |
|  | Age, Sex, GGT, TG, Attenuation | 0.657 (0.049) | 0.448 (0.065) |
|  | Age, Sex, GGT, TG, IDP | 0.684 (0.045) | 0.465 (0.071) |
| Type II diabetes | Age, Sex, Glucose | 0.914 (0.052) | 0.382 (0.130) |
|  | Age, Sex, Glucose, IDP | 0.923 (0.042) | 0.390 (0.129) |

Supplementary Fig. 1. The component composition of individuals diagnosed with metabolic syndrome in the baseline development cohort. HTN, hypertension; WC, waist circumference; HDL, high-density lipoprotein; TG, triglycerides.


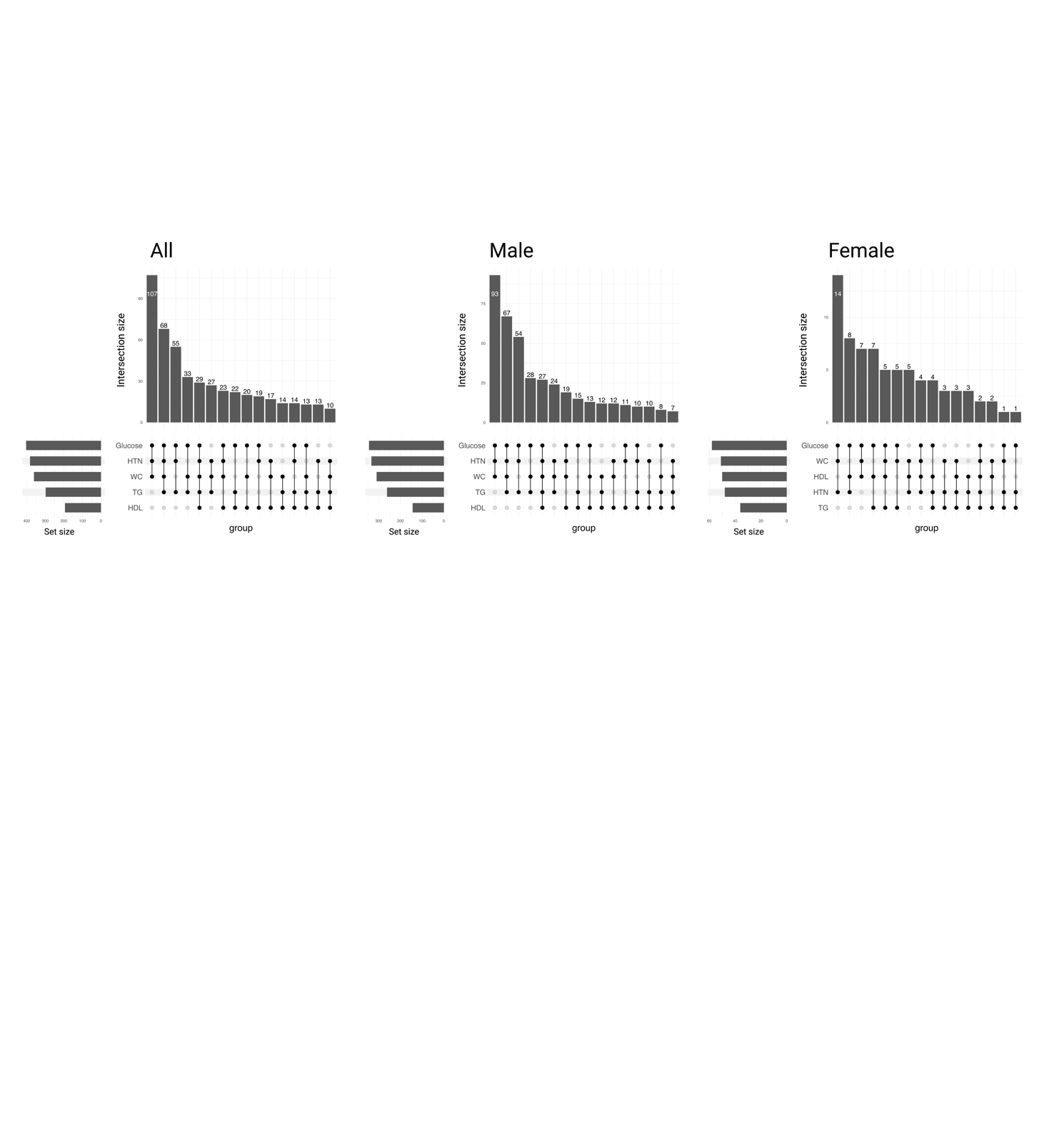


Supplementary Fig. 2. **a**, Overview of the 3D liver segmentation model and feature extraction procedure. Numbers shown about the convolution blocks are the number of channels and resulting features that are extracted from that block. **b**, Overview of 3D feature extraction model. The masked and cropped CT volume is input into the pretrained encoder, and the output of individual encoder blocks are extracted as the feature representation. **c,** Overview of the metabolic syndrome classifier. The feature representations for each patch are concatenated as input (size = 1120). There are two hidden layers (size = 512) followed by an attention pooling module to generate a volume level embedding. There is a final hidden layer (size = 256) before the classification of metabolic syndrome.


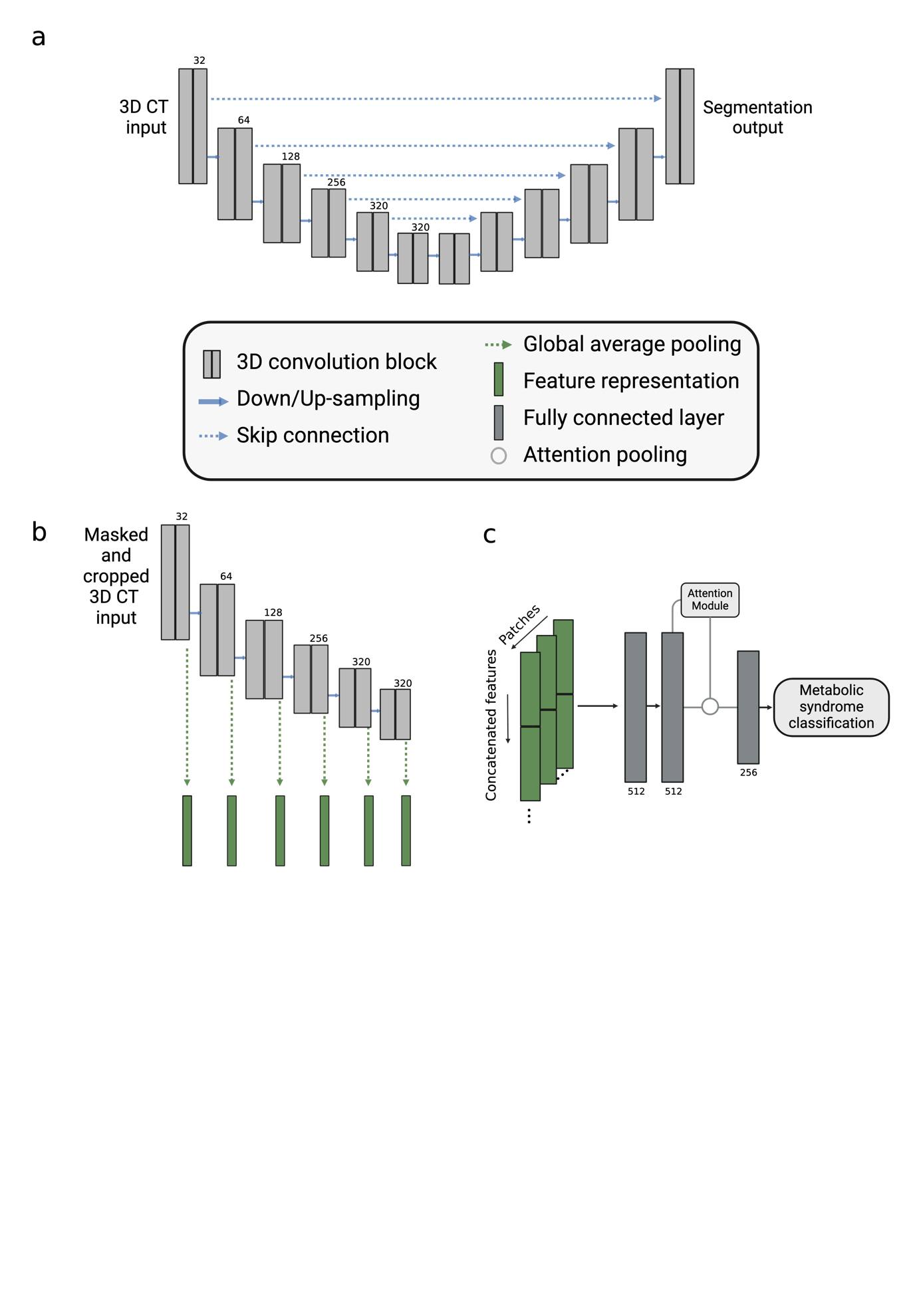


Supplementary Fig. 3. **a,** Predictive performance comparing covariates and attenuation to the IDP in the external validation cohort. All models include the covariates listed in the first column of the plots. **b**, Correlation between the IDP and metabolic syndrome risk factors. IDP, image-derived phenotype; AUC, area under receiver-operating curve; AUPRC, area under the precision-recall curve; Attenuation, median volumetric liver attenuation (expressed as Hounsfield units); VAT, visceral adipose tissue; SBP, systolic blood pressure; DBP, diastolic blood pressure; WC, waist circumference; Gluc, glucose; HDL, high-density lipoprotein; TG, triglycerides; **** *P* < 0.0001, paired t-test.


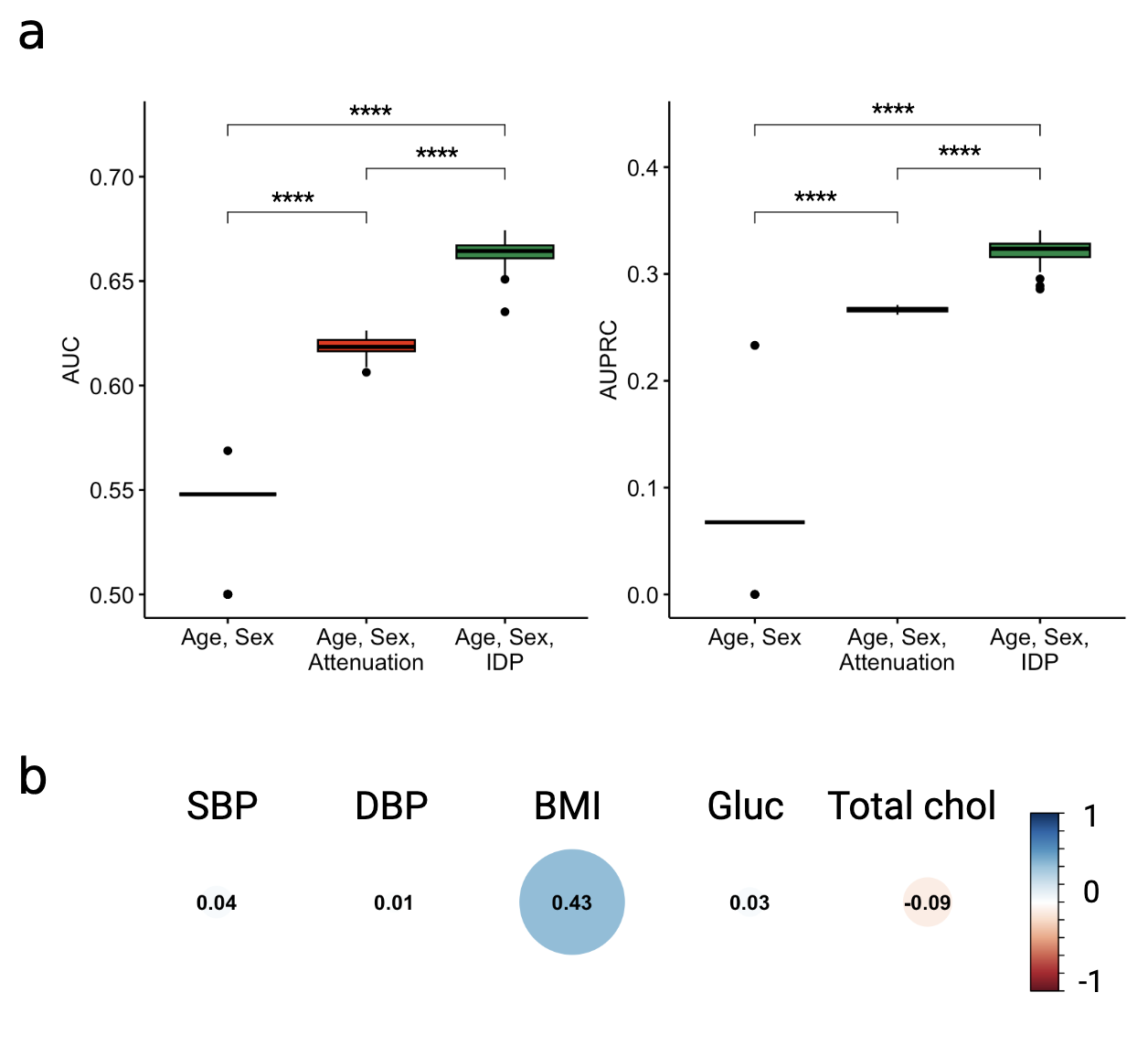


Supplementary Fig. 4. The baseline component composition of individuals who were not diagnosed with metabolic syndrome at baseline, however our model classified as metabolically abnormal, who went on to get disease. HTN, hypertension; WC, waist circumference; HDL, high-density lipoprotein; TG, triglycerides. Note that HTN is referring to blood pressure above a certain threshold, not hypertension diagnosis.


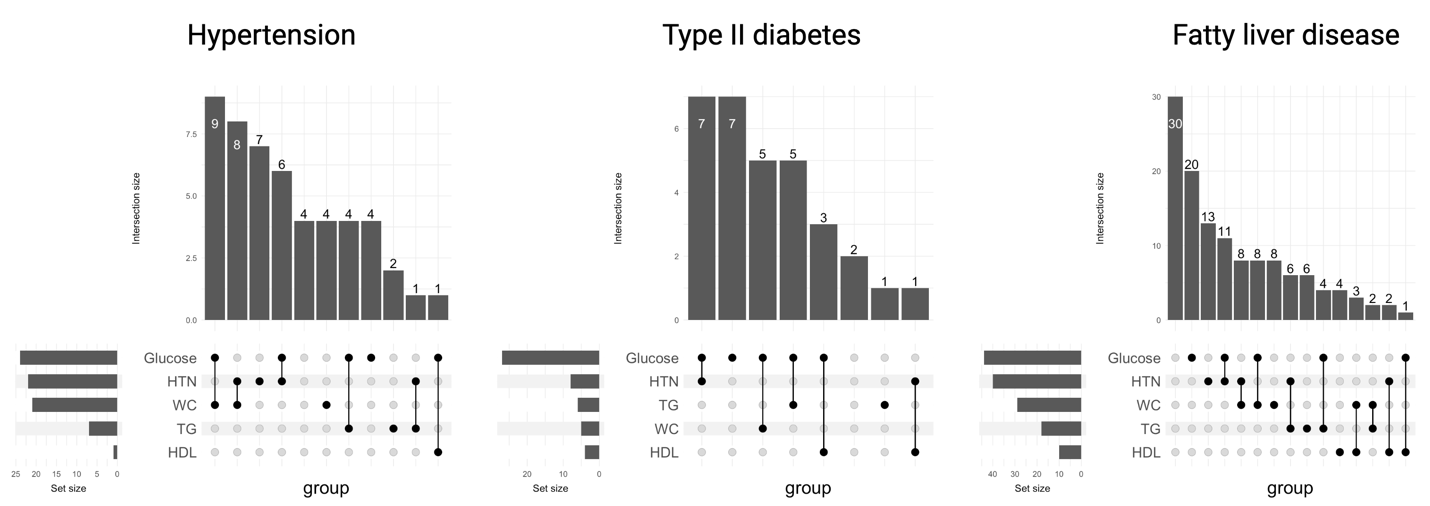

Supplement: Supplementary file 1 — Supplementary Material 1 [file 12967_2024_5163_MOESM1_ESM.docx]
